# Two tales: Worldwide distribution of Central Asian (CAS) versus ancestral East-African Indian (EAI) lineages of *Mycobacterium tuberculosis* underlines a remarkable cleavage for phylogeographical, epidemiological and demographical characteristics

**DOI:** 10.1371/journal.pone.0219706

**Published:** 2019-07-12

**Authors:** David Couvin, Yann Reynaud, Nalin Rastogi

**Affiliations:** WHO Supranational TB Reference Laboratory, Tuberculosis and Mycobacteria Unit, Institut Pasteur de la Guadeloupe, Abymes, Guadeloupe, France; Banaras Hindu University, INDIA

## Abstract

The East African Indian (EAI) and Central Asian (CAS) lineages of *Mycobacterium tuberculosis* complex (MTBC) mainly infect tuberculosis (TB) patients in the eastern hemisphere which contains many of the 22 high TB burden countries including China and India. We investigated if phylogeographical, epidemiological and demographical characteristics for these 2 lineages differed in SITVIT2 database. Genotyping results and associated data (age, sex, HIV serology, drug resistance) on EAI and CAS lineages (n = 10,974 strains) were extracted. Phylogenetic and Bayesian, and other statistical analyses were used to compare isolates. The male/female sex ratio was 907/433 (2.09) for the EAI group vs. 881/544 (1.62) for CAS (p-value<0.002). The proportion of younger patients aged 0–20 yrs. with CAS lineage was significantly higher than for EAI lineage (18.07% vs. 10.85%, p-value<0.0001). The proportion of multidrug resistant and extensively drug resistant TB among CAS group (30.63% and 1.03%, respectively) was significantly higher than in the EAI group (12.14% and 0.29%, respectively; p-value<0.0001). Lastly, the proportion of HIV+ patients was 20.34% among the EAI group vs. 3.46% in the CAS group (p-value<0.0001). This remarkable split observed between various parameters for these 2 lineages was further corroborated by their geographic distribution profile (EAI being predominantly found in Eastern-Coast of Africa, South-India and Southeast Asia, while CAS was predominantly found in Afghanistan, Pakistan, North India, Nepal, Middle-east, Libya, Sudan, Ethiopia, Kenya and Tanzania). Some geo-specificities were highlighted. This study demonstrated a remarkable cleavage for aforementioned characteristics of EAI and CAS lineages, showing a North-South divide along the tropic of cancer in Eastern hemisphere–mainly in Asia, and partly prolonged along the horn of Africa. Such studies would be helpful to better comprehend prevailing TB epidemic in context of its historical spread and evolutionary features, and provide clues to better treatment and patient-care in countries and regions concerned by these lineages.

## Introduction

Africa and Asia are the two continents concentrating the highest incidence of worldwide *Mycobacterium tuberculosis* complex (MTBC) cases. Despite the fact that incidence of tuberculosis (TB) is globally slowly declining every year, its impact remains important. Of the estimated 10.4 million incident cases of TB in 2015, more than half (61%) occurred in Asia. A further one quarter (26%) occurred in Africa, which also had the highest rates of cases and deaths relative to population [1]. In such a context, TB remains a priority communicable disease, even today, as underlined by the recent fact sheet from World Health Organization (WHO; [1]). Clearly, there is a need for a global concerted action to better monitor and control TB spread worldwide and foster relevant actions to prevent the disease. The changing face of TB epidemiology due to increased migration to escape wars, conflicts and poverty (as well as travel for leisure), should be ideally addressed using a multidisciplinary approach to address this worldwide scourge. Knowing that *Mycobacterium tuberculosis* and humans have co-evolved for thousands of years [2], such an objective can only be achieved using modern tools of molecular epidemiology in conjunction with international databases and web tools able to extract patient demographic and clinical data from longitudinal studies, allowing to identify and highlight the various ways of ongoing TB transmission and a better understanding of the disease.

Despite known limitations [3], classical genotyping methods such as CRISPR-based spoligotyping or MIRU-VNTRs are still helpful to confidently identify TB strains. Other methods such as IS*6110*-RFLP, Large Sequence Polymorphisms (LSP), single nucleotide polymorphisms (SNPs), and more recent Whole Genome Sequencing (WGS) are also important tools for identification of circulating MTBC clones [4–9]. Establishment of databases and web tools such as MIRU-VNTRplus [10], SITVIT WEB [11] or SITVIT2 [12], fill the needful among tools already available for epidemiological surveillance of TB. Using phylogenetic and Bayesian population structure analyses [13–16], recent studies could reveal geographical specificities of several MTBC lineages/sublineages such as Latin American and Mediterranean (LAM), T and Beijing; helping to understand prevailing TB epidemiology as well as to decipher the history and characteristics of disease propagation.

In the present study, based on the recently released SITVIT2 database [12], we describe the overall distribution and epidemiological features of spoligotyping-based East-African-Indian (EAI–also known as Lineage 1 or the LSP-based Indo-Oceanic lineage), and Central Asian (CAS–also known as LSP-based Lineage 3) lineages, which are predominantly found in Eastern Africa, South-East Asia, Indian subcontinent, and Western Asia [17]. Noting confusion in the literature on the use of terms EAI and CAS per LSP-based nomenclature [7], the readers are reminded that we hereby use these terms as spoligotyping-based signatures defined in the SITVIT2 database [12]. EAI and CAS lineages have been subdivided into several sublineages, and recent studies have highlighted potential existence of several geo-specific sublineages [18–19]. The present study aims to better characterize the genetic, demographic, evolutionary history and epidemiological features of CAS and EAI lineages.

## Materials and methods

### Ethics statement

All the data (summarized in **S1 and S2 Tables**) were collected from collaborating laboratories under the SITVIT2 database project [12], and duly de-identified prior to database entry. All demographic and clinical information was totally anonymized prior to analysis. The data is freely available from SITVIT2 and the cited literature.

### Database comparison and worldwide geographical distribution

Genotyping data were collected from the SITVIT2 proprietary database of the Institut Pasteur de la Guadeloupe [12] which is an updated version of the previously released SITVITWEB [11] database. Spoligotyping data on EAI and CAS lineage strains (n = 10,974 strains corresponding to 96 countries of patient origin) were extracted. We also retrieved associated MIRU-VNTR genotyping data available for 1650 isolates. Two formats of MIRU-VNTRs were analyzed: (i) the 12-loci format corresponded to MIRU loci in the order: MIRU 2, 4, 10, 16, 20, 23, 24, 26, 27, 31, 39 and 40; (ii) the 15-loci format corresponded to MIRU loci in the order: MIRU 4, 10, 16, 26, 31, and 40, ETRA, ETRC, QUB-11b, QUB-26, QUB-4156, Mtub04, Mtub21, Mtub30, and Mtub39. In this database, Spoligotype International Type (SIT) and a 12 or 15-MIRU International Type (12MIT or 15MIT) designate patterns shared by two or more isolates, whereas “orphan” designates patterns reported for a single isolate. Most of the analysis made use of 12-loci MIRU-VNTRs since data was more widely available. Because of the lack of 24-loci MIRU-VNTR information in this study, (around 100 isolates available in only two or three countries), 24-loci MIRU-VNTR analyses were excluded from the analyses. Further analyses using this marker should be performed in the future.

Worldwide distribution analyses were based on country (2 letter country codes according to http://en.wikipedia.org/wiki/ISO_3166-1_alpha-2), and macro-geographical (United Nations subregions, list available at: http://unstats.un.org/unsd/methods/m49/m49regin.htm) level; note that Russia was attributed a new subregion by itself (Northern Asia) instead of including it among the rest of Eastern Europe. We also used an in-house web tool (“SpolSimilaritySearch” available at: http://www.pasteur-guadeloupe.fr:8081/SpolSimilaritySearch [20]) to distinguish between widespread, specific and/or confined spoligotype patterns; to pinpoint patterns with large deleted blocks; as well as to provide with the country distribution patterns for each queried spoligotype [20]. Nonetheless, caution must be taken when considering global distribution and percentage by country since it may be affected by the representativeness of specific genotypes.

### Phylogenetical, Bayesian and statistical analyses

MLVA Compare software version 1.03 (Ridom and Genoscreen; http://www.ridom.de/) and BioNumerics software version 6.6 (Applied Maths, Sint-Martens-Latem, Belgium; http://www.applied-maths.com/bionumerics) were used to draw minimum spanning trees (MSTs) based on spoligotypes and MIRU-VNTRs patterns, in order to visualize evolutionary relationships between clinical isolates. SpolTools software (available through http://spoltools.emi.unsw.edu.au/) was used to draw Spoligoforest trees based on the Fruchterman-Reingold algorithm [21–22]. Contrary to the MSTs, Spoligoforest are directed and not necessarily connected graphs, which permit to highlight the evolutionary relationships between ascendant and descendant spoligotype patterns. Bayesian population analyses was performed on 12-loci MIRU-VNTR markers using STRUCTURE version 2.3 software [23] using admixture model and computation of posterior estimates for the parameters of interest based on Markov chain Monte Carlo (MCMC) algorithm. This analysis was run in 10 parallel MCMC for K populations ranging from 3 to 10 for EAI dataset and 1 to 6 for CAS dataset, with a burn-in of 100000 iterations and a run length of 10^6^ iterations. The most probable number of K of population was estimated by the delta K calculated by the Evanno method [24] in the program STRUCTURE HARVESTER] [25]. Q-matrix medians were then calculated from 10 replicates for K = 6 for EAI dataset and K = 3 for CAS dataset, by using the Greedy algorithm implemented in CLUMPP 1.1.2 software [26]. Finally, MST analyses were then run using BioNumerics software on both EAI and CAS dataset with sublineages identified by STRUCTURE using a cutoff of 0.6. Hunter-Gaston Diversity Index (HGDI) was calculated for both MIRU-VNTRs and spoligotyping patterns using V-DICE tool (http://www.hpa-bioinfotools.org.uk/cgi-bin/DICI/DICI.pl).

STATA version 12 (https://www.stata.com/) and R version 3.3.1 (https://www.R-project.org) were used for statistical analyses. Univariate analyses were performed using Pearson's Chi-square test and Fisher's Exact Test. Multivariate analyses were performed using Principal component analysis (PCA) from R packages "gmodels", "ggplot2" and "factoextra" (using the covariance matrix) [27–31]. Spoligotyping patterns were used as variables and the first two Principal Components which explain most of the variation were used to draw the PCAs in function of sublineages or origin country of patients. P values <0.05 were considered as statistically significant. Odds Ratios (OR) and 95% Confidence Intervals (CI) were calculated to better visualize the significance of the statistical tests.

## Results

### Global distribution maps

**Fig 1:**
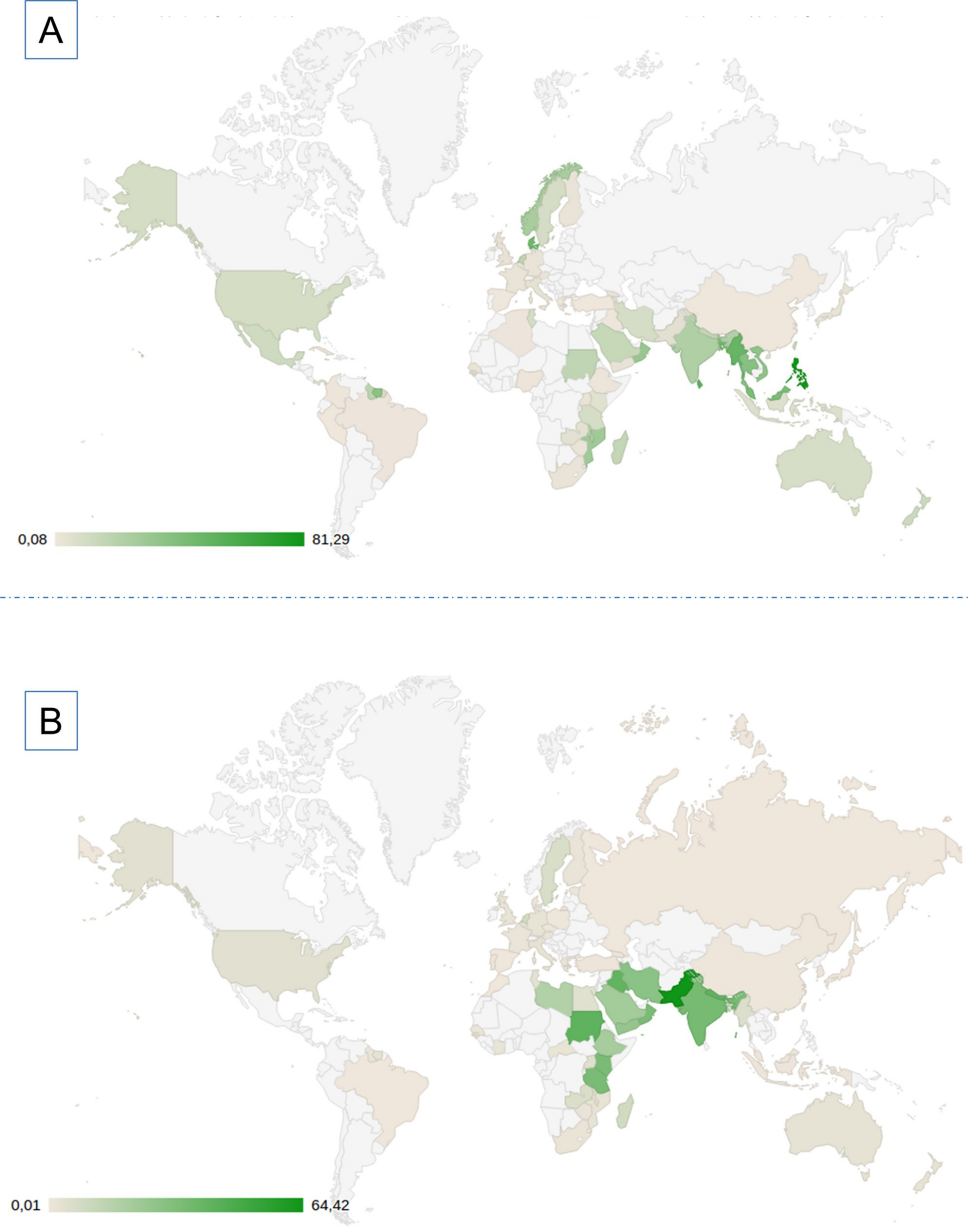
Global Map of distribution (percentage) of EAI (A) and CAS (B) lineages by country of isolation. For better clarification, users can directly and interactively visualize distribution of both EAI (L1) and CAS (L3) lineages on SpolSimilaritySearch’s “Geographical Distribution” web page (http://www.pasteur-guadeloupe.fr:8081/SpolSimilaritySearch/geo.jsp).

**Fig 2:**
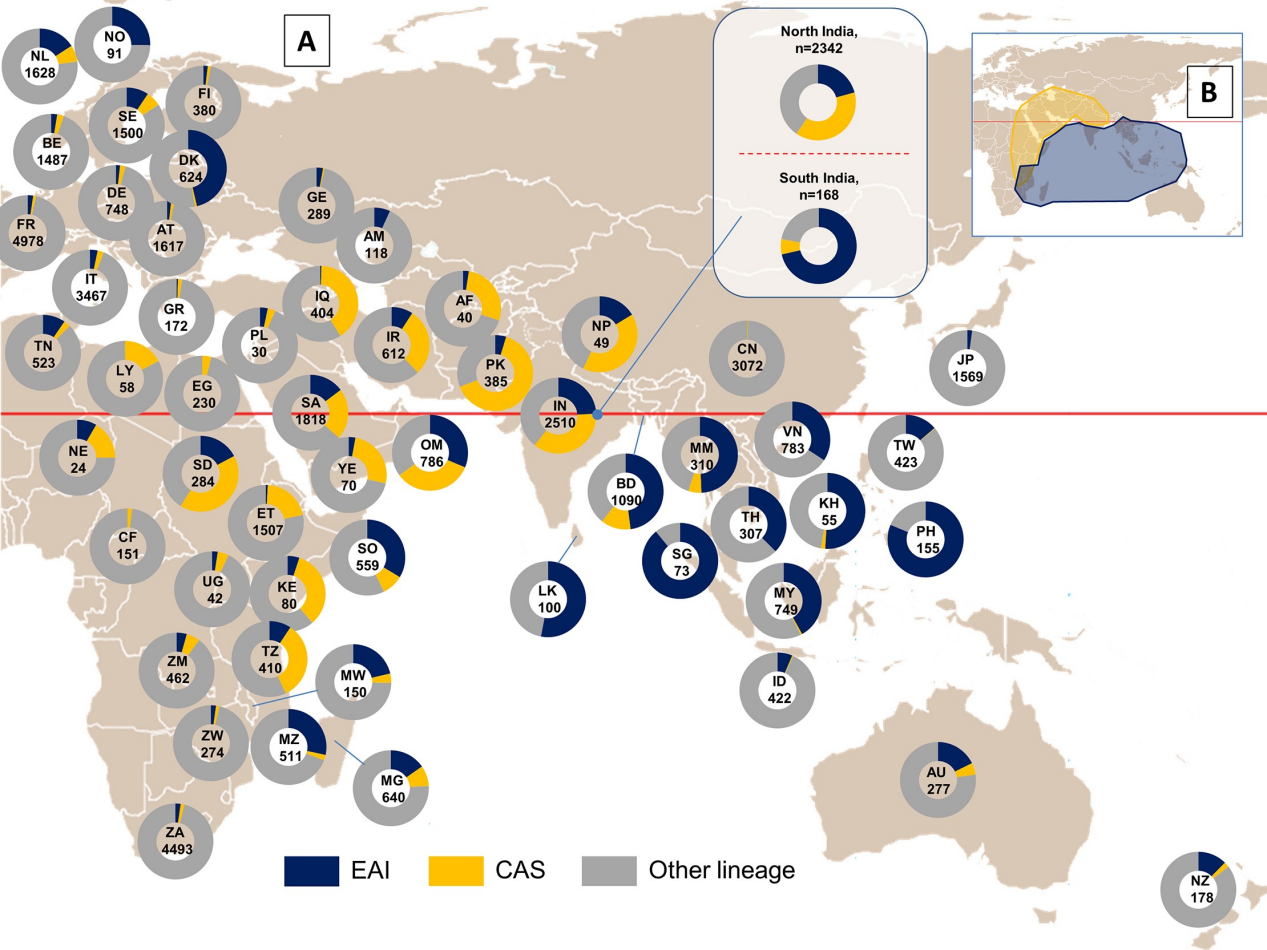
Distribution map of EAI and CAS vs. other MTBC lineages in Eastern hemisphere. (A) Map representing distribution of respective lineages taking in account proportions >2% for each lineage group (data based on country of isolation; when not available, the country of origin of patients such as for Afghanistan, Somalia, and Niger, was taken in account). Map freely available under Creative Commons License (http://en.wikipedia.org/wiki/Tropic_of_Cancer). Reprinted from https://en.wikipedia.org/wiki/Tropic_of_Cancer#/media/File:World_map_with_tropic_of_cancer.svg under a CC BY license, with permission from Mr. Laurent Cozic, original copyright 2009. (B) A smaller map in inset represents potential main dispersal areas of CAS (yellow) and EAI (blue) lineages.

Most cases of EAI and CAS lineages were concentrated in the Eastern hemisphere. EAI lineage strains were more common in East African, South and South-Eastern Asian countries, whereas CAS lineage strains were predominantly found in South and Western Asian countries, as well as in North- and East-African countries. Dissemination of EAI and CAS lineages in North America and Europe may have potentially occurred of late due to recent migration waves. Geographical maps were constructed to provide with an overview on the worldwide distribution of these two lineages in line with the data entered in the SITVIT2 database (**Figs 1 and 2**).

Regarding the distribution of EAI lineage stains, one can notice that:

- In countries boarding Indian and Pacific oceans, the highest proportion of EAI was found in the Philippines (81.29% of strains). The proportions of EAI were also remarkably high in neighboring countries as Vietnam (34.23%), Malaysia (41.92%), Myanmar (49.35%), Bangladesh (47.89%), Sri Lanka (53.00%), India (22.93%), Oman (31.30%), Mozambique (28.38%), and Madagascar (15.31%). EAI was present in a lesser extent in Indonesia and New Zealand (representing 6.40% and 12.36% respectively). A total of 17.25% of Sudanese strains belonged to EAI (**Fig 1**). Analyses based on country of origin of patients (**Fig 2**) highlighted the fact that EAI strains were considerably present in Somalia (33.63%). A smaller proportion of 5.88% EAI was observed in French Reunion Island. Moreover, 2.10% of EAI strains were reported in Japan, suggesting movements of populations from South East Asia to this country. Non-negligible proportions of EAI were also observed in Senegal (2.17%), Guinea-Bissau (5.44%) [32], and Tunisia (9.56%). The latter observations may be due to ancient or recent migration flows of populations inside Africa.
- Interestingly, EAI lineage strains were also found in the Americas (**Fig 1**), particularly in Mexico (11.75%), Trinidad and Tobago (12.50%), Guyana (18.68%), Suriname (35.71%), and French Guiana (9.61%). 9.68% of strains reported in the USA belonged to EAI. A non-negligible proportion of EAI was also found in Panama (3.91%). Globally, these observations corroborated recent analyses performed in these areas [33–34].
- The EAI lineage strains in Europe were mostly isolated from foreign-born patients, and more frequently reported in the North, accounting for 46.31% of strains isolated in Denmark, 25.27% in Norway, 9.40% strains in Sweden, and 1.84% in Finland [35]. On the other hand, EAI lineage strains accounted for less than 4% of strains in western and southern European countries, with the exception of the Netherlands (where they represented 15.85% of strains).

Regarding the distribution of CAS lineage stains, we may notice that:

- In the Indian subcontinent, the highest proportion of CAS was found in Pakistan with 64.42% of strains belonging to this lineage. CAS lineage was also well represented in Nepal (40.82%) and India (34.72%). However, this lineage was less frequently reported from Bangladesh (12.75%). On the other hand, CAS lineage was almost absent in Southeastern Asia, with the exception of Myanmar (where it represented 5.48% of isolates).
- In the case of Western Asia, the highest proportions of CAS strains were found in Iraq (40.59%), Oman (33.59%), Iran (28.76%), Afghanistan (28.00%), Yemen (25.71%), and Saudi Arabia (21.73%).
- In Africa, the highest proportion of CAS strains were found in former Sudan (41.55%), followed by Kenya (33.75%), Tanzania (33.66%), Ethiopia (20.77%), Libya (17.24%), Somalia (9.00%), and Madagascar (8.75%). CAS lineage strains were less noticeable in other African countries such as Zambia (5.84%), Uganda (4.76%), and Egypt (3.91%). Globally, CAS lineage strains were less common in Europe (7.13% of isolates in the Netherlands, 6.40% in Sweden, and less than 6% in other European countries), and the Americas (3.66% of strains in the US, and 2.68% of strains in Suriname); and mostly accountable to recent migrations from countries where such strains are endemic [36–37].

A certain synchronization of movements of EAI and CAS lineages is obvious (**Fig 2**), probably due to ancestral history of traditional African-Asian trading network and migrations [19]. Isolates belonging to CAS lineage were noticeably more confined and clustered than those belonging to EAI lineage (**Table 1 and Fig 2**). Moreover, several scenarios could be suggested to explain presence of these lineages in particular areas (a priori not due to recent peopling and migrations). Interestingly, the presence of EAI lineage was detected in Central and South-American countries such as Mexico, Guyana, Suriname and French Guiana. Ancient as well as recent peopling and migrations may explain the presence of this lineage in the Americas.

**Table 1:** Detailed description of the dataset used (n = 10974 strains).

	CAS (%)	EAI (%)
**Database**		
number of strains	4362	6612
number of countries (isolation)	68	77
number of countries (origin of patients)	52	92
**Spoligotyping**		
Clustered	4102 (94.04)	5919 (89.52)
Unique	260 (5.96)	693 (10.48)
**Gender**		
Male	881 (61.82)	907 (67.69)
Female	544 (38.18)	433 (32.31)
M/F sex ratio	1.62	2.09
**Drug resistance**		
Pansusceptible	832 (57.14)	966 (69.80)
Any resistance (excluding MDR/XDR)	163 (11.20)	246 (17.77)
MDR	446 (30.63)	168 (12.14)
XDR	15 (1.03)	4 (0.29)
**HIV status**		
HIV+	15 (3.46)	36 (20.34)
HIV-	418 (96.54)	141 (79.66)
**Age groups**		
0–20 yrs.	249 (18.07)	87 (10.90)
21–40 yrs.	701 (50.87)	364 (45.61)
41–60 yrs.	302 (21.92)	242 (30.33)
>60 yrs.	126 (9.14)	105 (13.16)

### Bayesian population structure analysis of 12-loci MIRU-VNTRs

**Fig 3:**
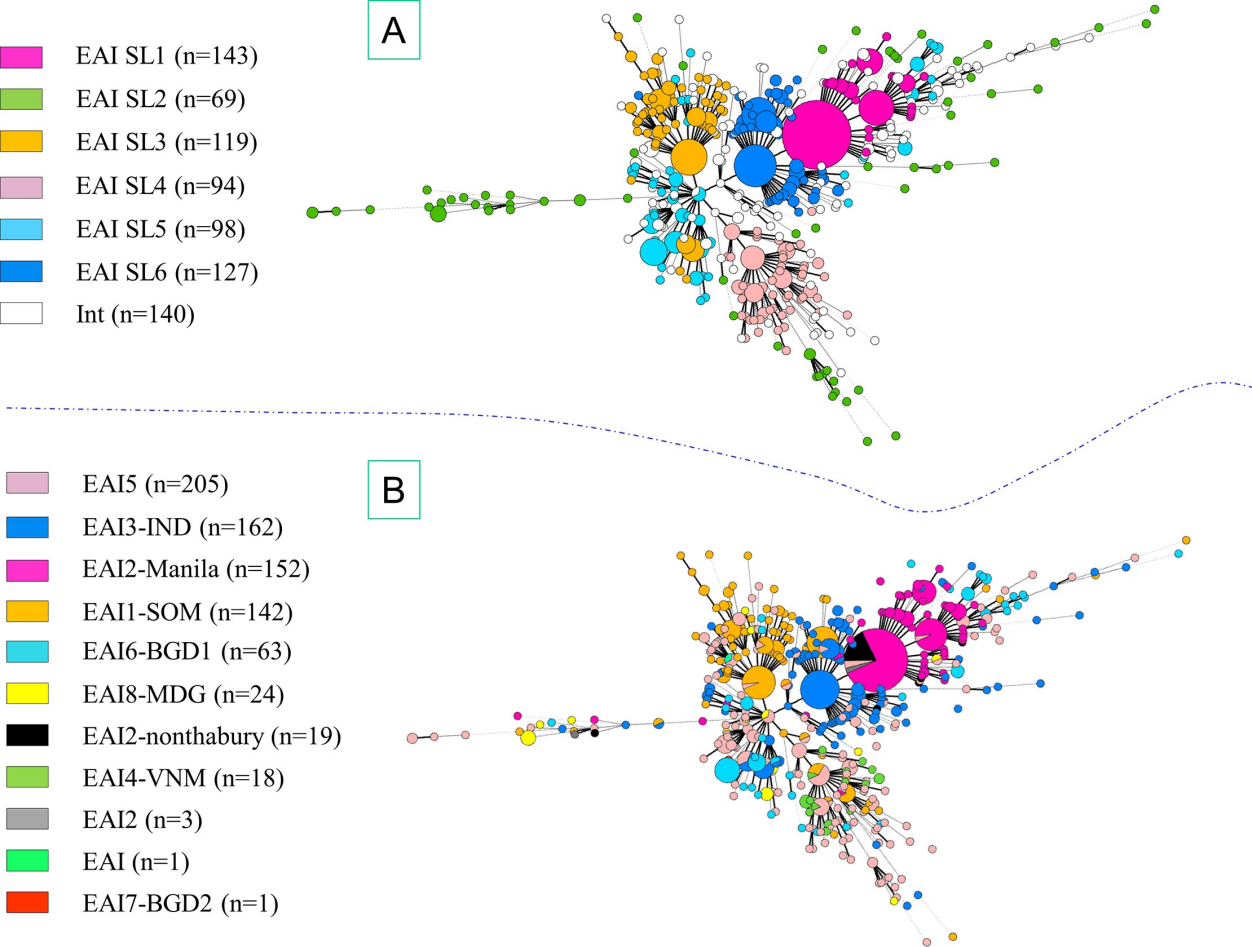
Minimum Spanning Trees (MST) based on 12-loci MIRU-VNTRs available for EAI lineage strains. Size of nodes (circles) representing genotypes were proportional to the number of strains. MST based on Bayesian population structure analysis (A). MST based on EAI sublineages according to SITVIT2 (B).

**Fig 4:**
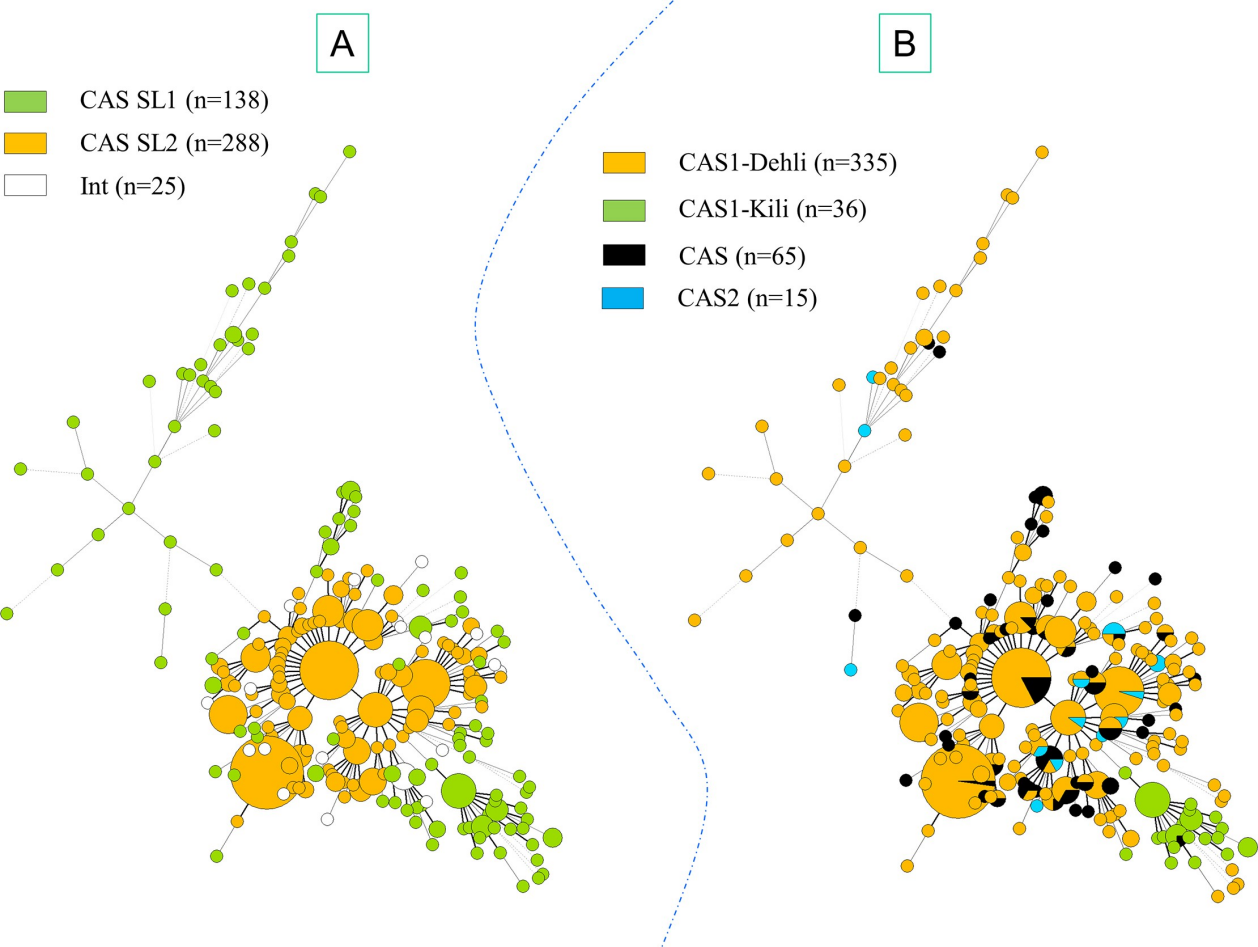
Minimum Spanning Trees (MST) based on 12-loci MIRU-VNTRs available from CAS lineage strains. Complexity of lines depends on the degree of correlation between two genotypes. Size of nodes (circles) representing genotypes were proportional to the number of strains. MST based on Bayesian population structure analysis (A). MST based on CAS sublineages according to SITVIT2 (B).

MSTs based on 12-loci MIRU-VNTRs for EAI and CAS lineage strains are illustrated in Figs 3 and 4 respectively. Bayesian population structure analyses provided relevant insights on the diversity of CAS and EAI lineages; briefly, with the exception of sublineage SL2 which constituted a loosely-knit group split into several branches in the MST analysis, the other 5 sublineages described were relatively well structured (**Figs 3 and 4**). When comparing this classification with spoligotyping, great convergences were outlined for 3 sublineages: SL1 corresponded mainly to EAI2-Manila (121/143) and in a lesser measure to EAI2-nonthaburi (15/143), SL3 to EAI1-SOM (81/119) and EAI5 (24/119), and finally SL6 corresponded to EAI3-IND (96/127) and EAI1-SOM (20/127); Congruence between Bayesian analysis and spoligotyping was less obvious for other sublineages: SL4 corresponded to EAI5 (59/94) but also to EAI4-VNM (17/94) and EAI1-SOM (14/94), SL5 was represented by EAI5 (42/98), EAI6-BGD1 (28/98), EAI3-IND (14/98) and EAI8-MDG (11/98).

Performing same analyses on CAS dataset led to subdivision into 2 sublineages (**Fig 4**): SL1 possibly corresponds to CAS1-Dehli (75/138) and CAS1-Kili (36/138), while SL2 undoubtedly corresponded to CAS1-Dehli (237/288). On the other hand, MSTs based on 12-loci MIRU-VNTRs of CAS (**S1 Fig**) and EAI (**S2 Fig**) lineage strains according to countries of origin of patients were unable to reveal a straightforward geographical specificity/variability within lineages that could be tentatively linked to a specific country of origin.

### Analyses based on spoligotyping and 12-loci MIRUs

EAI lineage strains (n = 6612) were represented by 1122 distinct spoligotyping patterns (n = 693 orphans and 5919 strains grouped in 428 SITs), while CAS lineage strains (n = 4362) were represented by 448 distinct spoligotyping patterns (n = 260 orphans and 4102 strains grouped in 188 SITs). Among the 820 EAI lineage strains with information on 12-loci MIRU-VNTRs, 513 possessed 12-MIT number. Similarly, among the 468 CAS lineage strains with information on 12-loci MIRU-VNTRs, 338 possessed 12-MIT number. The Neighbor-joining trees based analyses on spoligotyping patterns and 12-loci MIRU having a 12-MIT number for both CAS and EAI lineage strains are illustrated in Supplemental material (**S3 and S4 Figs**). As can be seen, the SIT/12-MIT couples were rather well subdivided in the trees drawn for both CAS and EAI lineage strains. Regarding the main genotypes belonging to CAS and EAI lineages, we have attempted to represent available information on couples of SIT/12-MIT and SIT/15-MIT, as well as the mean age, male/female sex ratio, and HIV+/HIV- status ratio of patients in **S5 Fig**. In this figure, a link was drawn when SIT/MIRU genotypes were associated (size of associated MIRUs was roughly proportional to number of genotypes; main changes between MIRU-VNTRs loci were shown in red).

Spoligotyping patterns belonging to EAI lineage (n = 6612) provided a HGDI of 0.970 (95% CI: 0.968–0.972), and spoligotypes belonging to CAS lineage (n = 4362) provided a HGDI of 0.869 (95% CI: 0.860–0.877); 12-loci MIRU-VNTRs patterns belonging to CAS lineage (available for more than 400 strains) provided a HGDI of 0.976 (95% CI: 0.969–0.983), and 12-loci MIRU-VNTRs patterns belonging to EAI lineage (available for more than 700 strains with no missing loci) provided a HGDI of 0.984 (95% CI: 0.979–0.988); 15-loci MIRU-VNTRs patterns belonging to CAS lineage (available for more than 200 strains with no missing loci) provided a HGDI of 0.958 (95% CI: 0.939–0.976), and 15-loci MIRU-VNTRs patterns belonging to EAI lineage (available for approximately 100 strains with no missing loci) provided a HGDI of 0.987 (95% CI: 0.977–0.996). HGDI indices provided significant discrimination power for both spoligotyping and 12-loci MIRU-VNTRs regarding CAS and EAI.

Some spoligotyping profiles seemed to be specific to delimited geographic areas. Spoligoforest analysis (hierarchical layout) and geographic distribution of patterns available from SpolSimilaritySearch [20] provided interesting insights on potential specific trends on evolutionary relationships and networking of patterns. Interestingly, SIT1198/CAS1-Delhi (which is similar to SIT25/CAS1-Delhi) appeared to be relatively well-spread in Iraq (26.32%), Sudan (21.05%), and Iran (15.79%) according to global distribution in SITVIT2. Regarding distribution in individual countries, we noted that SIT25 was more prevalent in Sudan (24.30%), Libya (15.52%), Ethiopia (9.56%), and Iraq (7.18%). Political, geographical, and historical relations between countries of Northeast Africa and Middle East (https://en.wikipedia.org/wiki/Foreign_relations_of_Sudan) should not be overlooked when focusing on the spread of specific CAS1 sublineage in this area.

When looking simultaneously at the genotyping distribution (Spoligoforest) and the geographical distribution, we can notice a potential trend of evolution of CAS1-Delhi beginning from India and Pakistan, and going to Middle East (Iran, Iraq, Saudi Arabia), until Eastern Africa (Sudan, Ethiopia). SIT21/CAS1-Kili predominant in Tanzania, seemed to have evolved from SIT26 *via* SIT22/CAS1-Delhi, frequently found in Saudi Arabia, Iraq, Oman, and Iran (representing respectively 32.61%, 21.74%, 15.22%, and 6.52 at a global scale). SIT22 was also sporadically present in Tanzania and Uganda (globally representing 3.26% and 1.09% of strains respectively in these countries), however, SIT22 has not been found in Sudan or Ethiopia. This observation may suggest the existence of various pathways of evolution for CAS lineage in East Africa. SIT22 and SIT25 could be tentatively re-labeled as CAS1-Middle_East and CAS1-Sudan respectively. However, further analysis will be needed to confirm this point. The Spoligoforest analysis performed (using CAS and EAI lineage SITs) emphasized the fact that CAS spoligotypes were more condensed/confined than EAI spoligotypes which were significantly more dispersed (**S6 and S7 Figs**).

The Spoligoforest of SITs of EAI lineage showed several ramifications/subgroups with well-known SITs constructing the central nodes such as SIT19/EAI2-Manila, SIT11/EAI3-IND, SIT48/EAI1-SOM, SIT139/EAI4-VNM, SIT109/EAI8-MDG, and SIT591/EAI6-BGD1 [17]. Interestingly, other less known ramifications/subgroups were also visible: such as SIT292, SIT1340, and SIT806. SIT1340/EAI6-BGD1 could be relabeled EAI6-Guiana because it represented respectively 16.07%, 6.59%, and 1.89% in Suriname, Guyana, and French Guiana. SIT1340, which was already mentioned in a paper by Millet et al. (2014) [33], appeared to be specific to the Guiana belt. Interestingly, both SIT1340 and SIT292/EAI6-BGD1 were characterized by the absence of spacers 23 and 37 (**S8 Fig**). The SIT292 (which is predominantly found in Myanmar and Bangladesh, representing respectively 9.03% and 7.52% of isolates in these countries), could be potentially relabeled as EAI6-MMR. Another pattern, SIT129/EAI6-BGD1, described in a recent paper by Conceição et al. (2017) [38], seemed to be specific to Mozambique. SIT2934/EAI2-Manila was predominantly linked to 12-MIT1154 (**S4 Fig**), and this spoligotyping pattern was found exclusively in Trinidad and Tobago.

**Fig 5:**
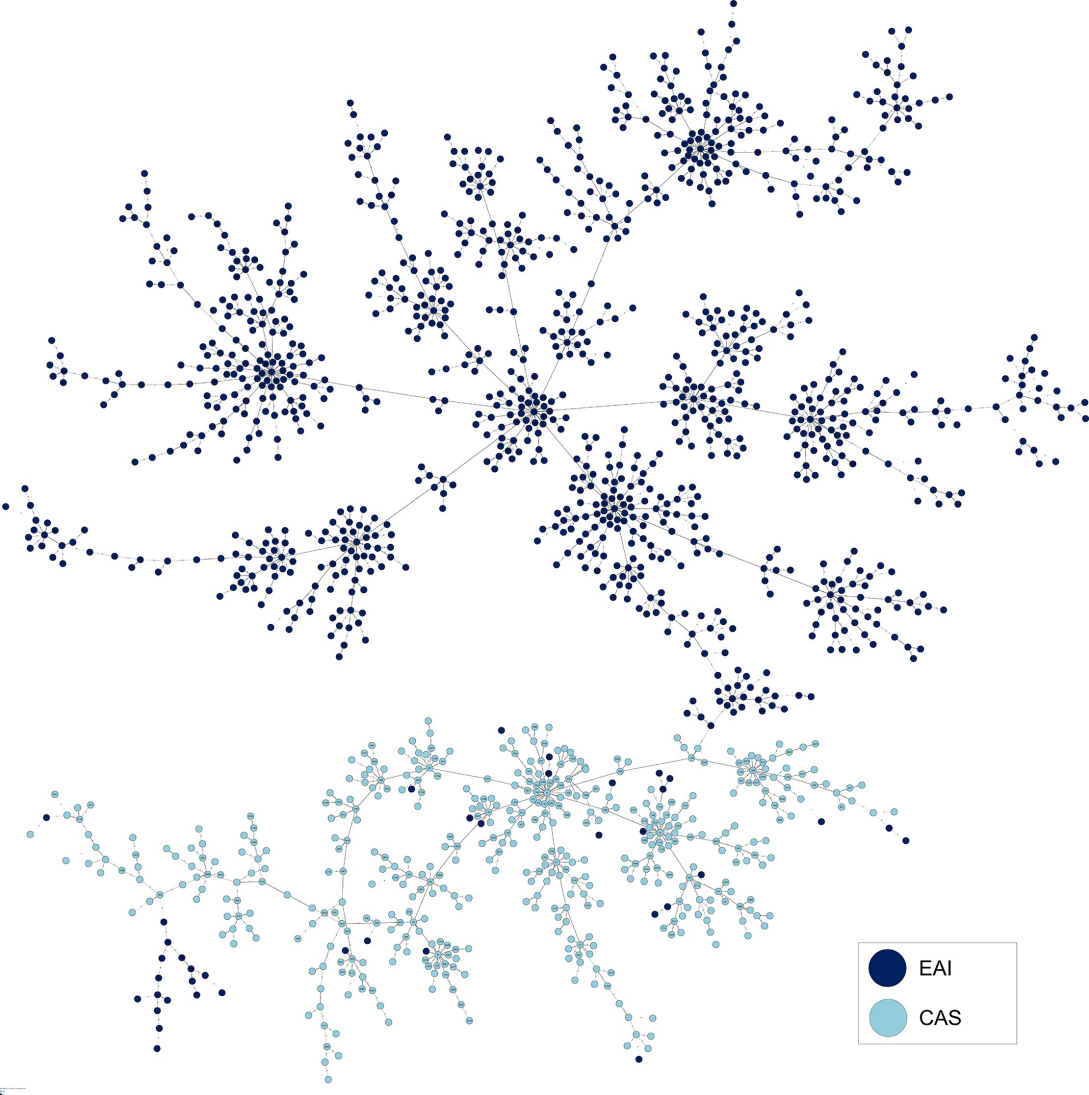
Spoligotyping-based MST for CAS and EAI lineage strains respectively. Nodes (circles) represent individual genotypes (note that in this figure, the size of the nodes is not proportional to the number of strains). Branches separating circles indicate the number of changes between spoligotypes.

The MST based on spoligotyping data (**Fig 5**) showed a significant split between genotypes belonging to CAS and EAI lineages. Spoligotypes of EAI group were mostly located on the top of the tree, whereas spoligotypes belonging to CAS were located on the bottom of the tree. Some main spoligotypes (identifiable by their SIT) were visible as central nodes forming star-shaped complexes (or sub-networks) with other related patterns. These patterns were often those representing main patterns such as SIT26/CAS1-Delhi and SIT19/EAI2-Manila. Among the rare spoligotyping patterns belonging to EAI lineage, yet appearing close to CAS group spoligotypes, one could mention SIT2963/EAI5 (found exclusively in Sudan). This spoligotype was classified as belonging to EAI lineage but was very similar to SIT25 which belongs to CAS1-Delhi, and was tentatively reclassified as CAS1-Sudan. Indeed, SIT2963 differs from SIT25 simply on the basis of the presence of spacer 33; it is noteworthy that the presence of spacer 33 is a criterion to distinguish EAI from CAS. Nonetheless, 12-loci MIRU-typing showed that SIT2963 was mainly associated with 12-MIT271 (226425153533) and 12-MIT1159 (226415153533), observations that underlined its potential link to the CAS lineage. Another rare pattern SIT1400/EAI5, identifiable by the absence of spacers 4 to 24 (located on the bottom left in the MST shown on **Fig 5**), was exclusively found in Bangladesh. Thus, spoligotyping-based MST analysis (**Fig 5**) allowed to identify potential patterns which may have been misclassified as EAI (probably because of typing error) or patterns which may be the result of mixed infection. Note that further analyses will be needed to clarify such cases, in order to correct potentially misclassified patterns in SITVIT2 database.

### Principal component analyses

Principal Component Analyses (PCAs) based on sublineages highlighted the fact that spoligotyping patterns belonging to EAI lineage were more diversified than spoligotypes belonging to CAS lineage (**S9 and S11 Figs**). One may notice that, among EAI group, spoligotypes belonging to sublineage EAI5 were particularly disorganized (localized in various areas of the plan) whereas other spoligotypes belonging to more specific EAI sublineages were well grouped, and mainly localized in a given area (e.g. EAI3-IND spoligotypes were predominantly found on the top left of the PCA). Regarding spoligotypes belonging to CAS lineage, one can also note a wide diversity observed for CAS (or CAS-like) spoligotypes as compared to CAS1-Kili and CAS2 sublineage spoligotypes that were relatively well clustered/grouped. One may further notice that spoligotypes belonging to CAS1-Delhi sublineage formed approximately three distinct groups concentrated on the right side of the plan. PCAs based on country of origin of patients (**S10 and S12 Figs**) revealed potential paths of TB dispersal in neighboring countries.

### Gender and age of patients

Noticeable differences could be observed regarding distribution of CAS and EAI lineages strains (globally and by subregions) in function of age-groups of the patients (i.e. 0–20 yrs, 21–40 yrs, 41–60 yrs, and >60 yrs). As one may have expected, the proportion of patients belonging to age group 21–40 yrs was higher for both CAS and EAI lineages (**S3 Table**) in the majority of subregions (except East Asia where proportion of patients from age group >60 yrs was predominant, representing 57.14% of patients). Within the EAI lineage group, the proportion of patients in age-group 0–20 yrs was higher or equal to 10% in AMER-C, AFRI-E, AFRI-N, EURO-N, ASIA-S, EURO-S, AFRI-W, CARIB, and ASIA-W (with percentages ranging from 10%-16.67%). Within the CAS lineage group, the proportion of patients in age-group 0–20 yrs was remarkably high in ASIA-W (14.79%), in AFRI-E (22.33%) and ASIA-S (22.63%). Since neither CAS nor EAI are endemic lineages in Europe [11, 12], cases reported from European areas were largely due to foreign-born patients originating from African and Asian countries.

### Drug resistance

Drug susceptibility testing (DST) information revealed that the proportion of multidrug resistant (MDR) and extensively drug resistant (XDR) TB strains among CAS group (30.63% and 1.03%, respectively) was significantly higher than in the EAI group (12.14% and 0.29%, respectively; p-value<0.0001). However, the proportion of MTBC strains presenting any resistance was relatively higher among EAI group (17.77%) as compared to CAS group (11.20%). Furthermore, the proportion of pansusceptible strains was higher among EAI lineage (69.80%) as compared to CAS lineage (57.14%) (**Table 1**). Time series data representing the evolution of drug resistance for both CAS and EAI lineages during 1990–2011, showed that MDR, XDR, and any drug resistance proportions were more and more visible from 2004 onward. Particularly high proportions of MDR-TB strains were recorded for CAS (48.24%) and EAI (28.72%) in 2009 (**S13 Fig**).

### HIV serology

The proportion of HIV+ patients was 20.34% among the EAI group vs. 3.46% in the CAS group (p-value<0.0001; Odds Ratio 0.14, 95%CI [0.07–0.27]). This observation was also corroborated by data on worldwide distribution of HIV: EAI lineage strains were mostly associated with HIV positive patients potentially because of the fact that this lineage was mostly localized in geographic areas where HIV epidemic is more prevalent (such as Sub-Saharan Africa and Southeast Asia), whereas HIV serology was less prevalent in Central Asia and Middle East (https://www.avert.org/global-hiv-and-aids-statistics).

## Discussion

Diversities of CAS and EAI lineages, notably the EAI lineage, could be concomitantly explained by historical factors, human migrations, and trade. The significant heterogeneity of EAI lineage may reflect scenarios of its successive introduction and adaptations to particular populations and/or ethnic groups. An example of similar heterogeneity is also reflected by the huge number of languages spoken by people in areas of AFRI-E or ASIA-SE (inside one given country, the number of spoken languages could exceed hundreds, as well as the number of ethnic groups that may exist). Thus, the propagation of CAS and EAI lineage strains could have been due to numerous factors that had a link with ancient and recent human history in the respective areas. Displacement of populations and peaks of empires/kingdoms interacting with various populations/civilizations may have greatly impacted dissemination and particular (co-)evolution of these specific lineages within the hosts during time in the southern eastern hemisphere. Furthermore, the spread of both CAS and EAI lineages may have been influenced by ancient African-Asian trade networks (http://castinet.castilleja.org/users/pmckee/africaweb/swahilistates.html) as well as the routes of the Silk Road (http://en.wikipedia.org/wiki/Silk_Road) as described in a recent study corroborating historical influences in TB spread in Africa and Eurasia [39]. Several traits of historical movements of populations have certainly played a role in TB spread. Other migrations occurring inside Africa may also have fostered spread of EAI or CAS lineages in North Africa for example (due to people trying to escape poverty and political turmoil from Sub-Saharan Africa).

Regarding the Indian subcontinent, EAI lineage strains are mostly found in the south part of India, whereas CAS lineage strains are predominantly present in the north, as recently underlined [40]. Data obtained during routine surveillance of verified MTBC cases reported in the Netherlands between 1993 and 2011 [41], suggested that on average, cluster's propensity to propagate (CPP) values from Euro-American lineage strains were higher than Beijing and EAI strains. Furthermore, this study [41] suggested that CPP values from strains belonging CAS lineage were on average higher than EAI strains.

The fact that EAI lineage strains were detected in Mexico suggested that this lineage may have been imported from Asia through the Bering Strait [42], and disseminated among Native Americans. More recent patterns of people displacement may also have played important roles in the spread of EAI lineage strains in South America and the Caribbean. One may name the displacement of Hmong people (https://en.wikipedia.org/wiki/Hmong_people) from Southeast Asia to French Guiana as a potential cause for presence of some EAI lineage strains in this area. TB spread and persistence have a certain link with poverty and social features of some regions and countries. As we may know, life expectancy is unequally distributed among countries (https://en.wikipedia.org/wiki/List_of_countries_by_life_expectancy). Hence, the relevant proportions (>10% of cases) of CAS and EAI lineage strains affecting young people (0–20 yrs) in certain areas of Africa and Asia, even in Central America or the Caribbean, represent an alarming cause for concern. Since emergence of drug resistance, political turmoil, poverty and armed conflicts lead patients to seek care in other countries [43], concerted efforts and actions directed towards policies reducing inequalities between countries and populations at global as well as country or regional levels are most urgently needed. One should also find ways to enhance treatment capacities of developing countries, particularly those from southeastern hemisphere.

Briefly, this study focusing on CAS and EAI lineages aimed to better understand phylogeographical, epidemiological and demographical trends of these 2 lineages. Relevant cleavages were remarkably visible when comparing proportions of HIV+ patients and MDR or XDR-TB. Recent genotyping studies showed that CAS lineage strains appeared to be closely related to Beijing family (lineage 2) strains. Regarding Spoligoforest analyses, we noticed that CAS genotype (better exemplified by SIT26/CAS1-Delhi) was shown as a potential “precursor” of Beijing genotype [44, 36]. One may notice that the geographical area in which CAS lineage is predominantly found is never far from China and its neighboring regions, where the Beijing lineage potentially evolved/originated from CAS strains.

Finally, the diversity concerning the EAI lineage is visibly best represented through sublineage EAI5 (or EAI-like which represents basic strains belonging to this lineage). This extraordinary diversity suggests that other EAI sublineages yet remain to be discovered. Nonetheless, caution must be taken when analyzing isolates belonging to EAI5 because misclassifications or mixed infection may occur.

Specificities of CAS and EAI lineages have been reported in other studies [45–48] [18–19]. These observations may indicate that other specificities concerning these lineages could be found in future analyses. However, further analyses using more recent techniques (such as Whole Genome Sequencing) will be needed to better highlight features of both EAI and CAS lineages. Phylogenetical analyses associated with geographic distributions allowed suggestions of potentially new sublineages for CAS and EAI (CAS1-Middle_East, CAS1-Sudan, EAI6-MMR, or EAI6-Guianas). Although spoligotyping present several known weaknesses, it allowed to robustly assigning lineages [49]. Other approaches (such as WGS and SNPs) may be utilized to better estimate recent transmission events [49]. Two recent studies have notably used SNP-typing for a more robust lineage classification [50–51]. Despite known limitations, MIRU-VNTR and Spoligotyping remain more feasible and affordable methods that should not be thrown away but used in conjunction with new generation methods. Furthermore, a recent study showed the efficacy of spoligotyping data to analyze geospatial distribution of MTBC strains in Africa [52], drawing new insights into specificities of TB epidemics and TB spread in the continent. Another recent study highlighted genetic diversity of MTBC strains in Botswana using spoligotyping and MIRU-VNTRs [53].

## Conclusion

This study highlighted a remarkable cleavage for phylogeographical, epidemiological and demographical characteristics of EAI and CAS lineages, showing a North-South divide along the tropic of cancer in Eastern hemisphere–mainly in Asia, and partly prolonged along the horn of Africa. Such studies would be helpful to better comprehend prevailing TB epidemic in context of its historical spread and evolutionary features, and provide clues to better treatment and patient-care in countries and regions concerned by these lineages, i.e., in the Indian subcontinent, South-East Asia, Middle-east, and East-Africa. Furthermore, international strategies for TB control such as DOTS (Directly Observed Treatment, Short Course) must be sustained, and efforts should be concentrated on areas presenting higher risks.

## Supporting information

S1 Fig MST based on 12-loci MIRU-VNTRs of CAS lineage strains, according to country of origin of patients. Size of nodes or complexity of lines are the same as in Figs 3 and 4.
(TIF)

S2 Fig MST based on 12-loci MIRU-VNTRs of EAI lineage strains, according to country of origin of patients. Size of nodes or complexity of lines are the same as in Figs 3 and 4.
(TIF)

S3 Fig Neighbor joining tree based on 338 CAS lineage strains containing information on both spoligotyping patterns and 12-loci MIRU having a 12-MIT number. Each leaf represents a SIT/12-MIT couple.
(TIF)

S4 Fig Neighbor joining tree based on 513 EAI lineage strains containing information on both spoligotyping patterns and 12-loci MIRU having a 12-MIT number. Each leaf represents a SIT/12-MIT couple.
(PNG)

S5 Fig Main genotypes of CAS and EAI lineage strains with information on spoligotypes (SIT), 12 and 15-loci MIRU-VNTRs, as well as mean age, male/female sex ratio, and HIV+/HIV- status ratio of patients. When SITs and/or MIRU-VNTRs genotypes were associated, a link was drawn. Size of associated MIRUs was roughly proportional to number of genotypes. Main changes between MIRU-VNTRs loci were shown in red.
(TIF)

S6 Fig Spoligoforest tree on CAS dataset drawn using by SpolTools software (available through http://spoltools.emi.unsw.edu.au/; [21–22], and shown as a Hierarchical Layout. Each spoligotype pattern from the study is represented by a node with area size being proportional to the total number of isolates with that specific pattern (number shown in brackets under the SIT number). Changes (loss of spacers) are represented by directed edges between nodes, with the arrowheads pointing to descendant spoligotypes. In this representation, the heuristic used selects a single inbound edge with a maximum weight using a Zipf model. Solid black lines link patterns that are very similar, i.e., loss of one spacer only (maximum weigh being 1.0), while dashed lines represent links of weight comprised between 0.5 and 1, and dotted lines a weight less than 0.5.
(PDF)

S7 Fig Spoligoforest tree on EAI dataset drawn using SpolTools software (available through http://spoltools.emi.unsw.edu.au/; [21–22], and shown as a Hierarchical Layout. Each spoligotype pattern from the study is represented by a node with area size being proportional to the total number of isolates with that specific pattern (number shown in brackets under the SIT number). Changes (loss of spacers) are represented by directed edges between nodes, with the arrowheads pointing to descendant spoligotypes. In this representation, the heuristic used selects a single inbound edge with a maximum weight using a Zipf model. Solid black lines link patterns that are very similar, i.e., loss of one spacer only (maximum weigh being 1.0), while dashed lines represent links of weight comprised between 0.5 and 1, and dotted lines a weight less than 0.5.
(PDF)

S8 Fig Maps representing potential geo-specificities of SITs 22, 25, 292 and 1340. (PDF)

S9 Fig PCA based on CAS lineage strains showing distribution of CAS sublineages in function of spoligotyping patterns. (PDF)

S10 Fig PCA based on CAS lineage strains showing country of origin of patients in function of spoligotyping patterns. (PDF)

S11 Fig PCA based on EAI lineage strains showing distribution of EAI sublineages in function of spoligotyping patterns. (PDF)

S12 Fig PCA based on EAI lineage strains showing country of origin of patients in function of spoligotyping patterns. (PDF)

S13 Fig Distribution (percentage) of drug resistance information for CAS and EAI lineages in function of year of isolation. (TIF)

S1 Table Global dataset used in this study for the CAS isolates. (XLS)

S2 Table Global dataset used in this study for the EAI isolates. (XLS)

S3 Table Distribution of age-groups of patients in function of lineages and UN subregions. (XLS)

## References

[1] World Health Organization (WHO) Global tuberculosis report 2016. http://apps.who.int/medicinedocs/documents/s23098en/s23098en.pdf

[2] Comas I, Coscolla M, Luo T, Borrell S, Holt KE, Kato-Maeda M, et al (2013) Out-of-Africa migration and Neolithic coexpansion of *Mycobacterium tuberculosis* with modern humans. Nat Genet 45:1176–1182. 10.1038/ng.2744 23995134 PMC3800747

[3] Comas I, Homolka S, Niemann S, Gagneux S (2009) Genotyping of genetically monomorphic bacteria: DNA sequencing in *Mycobacterium tuberculosis* highlights the limitations of current methodologies. PLoS One 4: e7815 10.1371/journal.pone.0007815 19915672 PMC2772813

[4] Kamerbeek J, Schouls L, Kolk A, van Agterveld M, van Soolingen D, Kuijper S, et al (1997) Simultaneous detection and strain differentiation of *Mycobacterium tuberculosis* for diagnosis and epidemiology. J Clin Microbiol 35:907–914. 9157152 10.1128/jcm.35.4.907-914.1997 PMC229700

[5] Supply P, Allix C, Lesjean S, Cardoso-Oelemann M, Rüsch-Gerdes S, Willery E, et al (2006) Proposal for standardization of optimized mycobacterial interspersed repetitive unit-variable-number tandem repeat typing of *Mycobacterium tuberculosis*. J Clin Microbiol 44:4498–4510. 10.1128/JCM.01392-06 17005759 PMC1698431

[6] van Embden JD, Cave MD, Crawford JT, Dale JW, Eisenach KD, Gicquel B, et al (1993) Strain identification of *Mycobacterium tuberculosis* by DNA fingerprinting: recommendations for a standardized methodology. J Clin Microbiol 31(2):406–9. 8381814 10.1128/jcm.31.2.406-409.1993 PMC262774

[7] Gagneux S, DeRiemer K, Van T, Kato-Maeda M, de Jong BC, Narayanan S, et al (2006) Variable host-pathogen compatibility in *Mycobacterium tuberculosis*. Proc Natl Acad Sci U S A 103(8):2869–73. 10.1073/pnas.0511240103 16477032 PMC1413851

[8] Hill V, Zozio T, Sadikalay S, Viegas S, Streit E, Kallenius G, et al (2012) MLVA based classification of *Mycobacterium tuberculosis* complex lineages for a robust phylogeographic snapshot of its worldwide molecular diversity. PLoS One 7(9):e41991 10.1371/journal.pone.0041991 22984400 PMC3439451

[9] Coll F, McNerney R, Guerra-Assunção JA, Glynn JR, Perdigão J, Viveiros M, et al (2014) A robust SNP barcode for typing *Mycobacterium tuberculosis* complex strains. Nat Commun 5:4812 10.1038/ncomms5812 25176035 PMC4166679

[10] Weniger T, Krawczyk J, Supply P, Niemann S, Harmsen D. (2010) MIRU-VNTRplus: a web tool for polyphasic genotyping of *Mycobacterium tuberculosis* complex bacteria. Nucleic Acids Res. 38(Web Server issue):W326–31. 10.1093/nar/gkq351 20457747 PMC2896200

[11] Demay C, Liens B, Burguière T, Hill V, Couvin D, Millet J, et al (2012) SITVITWEB–a publicly available international multimarker database for studying *Mycobacterium tuberculosis* genetic diversity and molecular epidemiology. Infect Genet Evol 12:755–766. 10.1016/j.meegid.2012.02.004 22365971

[12] Couvin D, David A, Zozio T, Rastogi N. (2018) Macro-geographical specificities of the prevailing tuberculosis epidemic as seen through SITVIT2, an updated version of the Mycobacterium tuberculosis genotyping database. Infect Genet Evol. pii: S1567–1348(18)30969-9. [Epub ahead of print] 10.1016/j.meegid.2018.12.030 30593925

[13] Reynaud Y, Millet J, Rastogi N. (2015) Genetic Structuration, Demography and Evolutionary History of *Mycobacterium tuberculosis* LAM9 Sublineage in the Americas as Two Distinct Subpopulations Revealed by Bayesian Analyses. PLoS One 10(10):e0140911 10.1371/journal.pone.0140911 26517715 PMC4627653

[14] Reynaud Y, Zheng C, Wu G, Sun Q, Rastogi N. (2017) Bayesian population structure analysis reveals presence of phylogeographically specific sublineages within previously ill-defined T group of *Mycobacterium tuberculosis*. PLoS One 12(2):e0171584 10.1371/journal.pone.0171584 28166309 PMC5293260

[15] Zheng C, Reynaud Y, Zhao C, Zozio T, Li S, Luo D, et al (2017) New *Mycobacterium tuberculosis* Beijing clonal complexes in China revealed by phylogenetic and Bayesian population structure analyses of 24-loci MIRU-VNTRs. Sci Rep 7(1):6065 10.1038/s41598-017-06346-1 28729708 PMC5519585

[16] Stucki D, Brites D, Jeljeli L, Coscolla M, Liu Q, Trauner A, et al (2016) *Mycobacterium tuberculosis* lineage 4 comprises globally distributed and geographically restricted sublineages. Nat Genet. 48(12):1535–1543. 10.1038/ng.3704 27798628 PMC5238942

[17] Brudey K, Driscoll JR, Rigouts L, Prodinger WM, Gori A, Al-Hajoj SA, et al (2006) *Mycobacterium tuberculosis* complex genetic diversity: mining the fourth international spoligotyping database (SpolDB4) for classification, population genetics and epidemiology. BMC microbiology 6:23 10.1186/1471-2180-6-23 16519816 PMC1468417

[18] Ismail F, Couvin D, Farakhin I, Abdul Rahman Z, Rastogi N, Suraiya S. (2014) Study of *Mycobacterium tuberculosis* complex genotypic diversity in Malaysia reveals a predominance of ancestral East-African-Indian lineage with a Malaysia-specific signature. PLoS One 9(12):e114832 10.1371/journal.pone.0114832 25502956 PMC4263714

[19] Mbugi EV, Katale BZ, Streicher EM, Keyyu JD, Kendall SL, Dockrell HM, et al (2016) Mapping of *Mycobacterium tuberculosis* Complex Genetic Diversity Profiles in Tanzania and Other African Countries. PLoS One 11(5):e0154571 10.1371/journal.pone.0154571 27149626 PMC4858144

[20] Couvin D, Zozio T, Rastogi N. (2017) SpolSimilaritySearch—A web tool to compare and search similarities between spoligotypes of *Mycobacterium tuberculosis* complex. Tuberculosis (Edinb). 105:49–52. 28610787 10.1016/j.tube.2017.04.007

[21] Tang C, Reyes JF, Luciani F, Francis AR, Tanaka MM (2008) SpolTools: online utilities for analyzing spoligotypes of the *Mycobacterium tuberculosis* complex. Bioinformatics 24:2414–415. 10.1093/bioinformatics/btn434 18710872

[22] Reyes JF, Francis AR, Tanaka MM (2008) Models of deletion for visualizing bacterial variation: an application to tuberculosis spoligotypes. BMC Bioinformatics 9:496 10.1186/1471-2105-9-496 19036166 PMC2620273

[23] Pritchard JK, Stephens M, Donnelly P. (2000) Inference of Population Structure Using Multilocus Genotype Data. Genetics; 155: 945–959 10835412 10.1093/genetics/155.2.945 PMC1461096

[24] Evanno G, Regnaut S, Goudet J. (2005) Detecting the number of clusters of individuals using the software structure: a simulation study. Mol Ecol. Blackwell Science Ltd; 14: 2611–2620. 10.1111/j.1365-294X.2005.02553.x 15969739

[25] Earl D, VonHoldt B. (2012) STRUCTURE HARVESTER: a website and program for visualizing STRUCTURE output and implementing the Evanno method. Conserv Genet Resour. Springer Netherlands; 4: 359–361

[26] Jakobsson M, Rosenberg NA. (2007) CLUMPP: a cluster matching and permutation program for dealing with label switching and multimodality in analysis of population structure. Bioinformatics; 23: 1801–1806 10.1093/bioinformatics/btm233 17485429

[27] Kassambara A. (2017) Practical Guide to Cluster Analysis in R: Unsupervised Machine Learning. ISBN-10: 1542462703 link: http://www.sthda.com/english/web/5-bookadvisor/17-practical-guide-to-cluster-analysis-in-r

[28] Wickham H. (2009). ggplot2: Elegant Graphics for Data Analysis. Springer-Verlag New York.

[29] Maechler M, Rousseeuw P, Struyf A, Hubert M, Hornik K. (2016). cluster: Cluster Analysis Basics and Extensions. R package version 2.0.5.

[30] Le S, Josse J, Husson F. (2008). FactoMineR: An R Package for Multivariate Analysis. Journal of Statistical Software, 25(1), 1–18. 10.18637/jss.v025.i01

[31] Galili T (2015). dendextend: an R package for visualizing, adjusting, and comparing trees of hierarchical clustering. Bioinformatics; 31(22):3718–20. 10.1093/bioinformatics/btv428 26209431 PMC4817050

[32] Groenheit R, Ghebremichael S, Svensson J, Rabna P, Colombatti R, Riccardi F, et al, (2011) The Guinea-Bissau family of *Mycobacterium tuberculosis* complex revisited. PLoS One 6(4): e18601 10.1371/journal.pone.0018601 21533101 PMC3080393

[33] Millet J, Baboolal S, Streit E, Akpaka PE, Rastogi N. (2014) A first assessment of *Mycobacterium tuberculosis* genetic diversity and drug-resistance patterns in twelve Caribbean territories. Biomed Res Int; 2014:718496 10.1155/2014/718496 24795893 PMC3985416

[34] Streit E, Baboolal S, Akpaka PE, Millet J, Rastogi N. (2015) Finer characterization of *Mycobacterium tuberculosis* using spoligotyping and 15-loci MIRU-VNTRs reveals phylogeographical specificities of isolates circulating in Guyana and Suriname. Infect Genet Evol. 30:114–9. 10.1016/j.meegid.2014.12.015 25528138

[35] Smit PW, Haanperä M, Rantala P, Couvin D, Lyytikäinen O, Rastogi N, et al (2013) Molecular epidemiology of tuberculosis in Finland, 2008–2011. PLoS One 8(12):e85027 10.1371/journal.pone.0085027 24386443 PMC3873426

[36] Fallico L, Couvin D, Peracchi M, Pascarella M, Franchin E, Lavezzo E, et al (2014) Four year longitudinal study of *Mycobacterium tuberculosis* complex isolates in a region of North-Eastern Italy. Infect Genet Evol 26:58–64. 10.1016/j.meegid.2014.05.004 24820340

[37] Pichat C, Couvin D, Carret G, Frédénucci I, Jacomo V, Carricajo A, et al (2016) Combined Genotypic, Phylogenetic, and Epidemiologic Analyses of *Mycobacterium tuberculosis* Genetic Diversity in the Rhône Alpes Region, France. PLoS ONE 11(4): e0153580 10.1371/journal.pone.0153580 27128522 PMC4851328

[38] Conceição EC, Rastogi N, Couvin D, Lopes ML, Furlaneto IP, Gomes HM, et al (2017) Genetic diversity of *Mycobacterium tuberculosis* from Pará, Brazil, reveals a higher frequency of ancestral strains than previously reported in South America. Infect Genet Evol; 56:62–74. 10.1016/j.meegid.2017.10.021 29081357

[39] O'Neill MB, Kitchen A, Zarley A, Aylward W, Eldholm V, et al, (2017) Lineage specific histories of *Mycobacterium tuberculosis* dispersal in Africa and Eurasia. bioRxiv 210161; 10.1101/210161 PMC6660993 31066139

[40] Singh J, Sankar MM, Kumar P, Couvin D, Rastogi N, Singh S (2015) Genetic diversity and drug susceptibility profile of *Mycobacterium tuberculosis* isolated from different regions of India. J Infect 71(2):207–19. 10.1016/j.jinf.2015.04.028 25934327

[41] Nebenzahl-Guimaraes H, Borgdorff MW, Murray MB, van Soolingen D (2014) A Novel Approach—The Propensity to Propagate (PTP) Method for Controlling for Host Factors in Studying the Transmission of *Mycobacterium tuberculosis*. PLoS One 9(5): e97816 10.1371/journal.pone.0097816 24849817 PMC4029888

[42] Bourgeon L, Burke A, Higham T (2017) Earliest Human Presence in North America Dated to the Last Glacial Maximum: New Radiocarbon Dates from Bluefish Caves, Canada. PLoS ONE 12(1): e0169486 10.1371/journal.pone.0169486 28060931 PMC5218561

[43] Cain KP, Marano N, Kamene M, Sitienei J, Mukherjee S, Galev A, et al (2015) The Movement of Multidrug-Resistant Tuberculosis across Borders in East Africa Needs a Regional and Global Solution. PLoS Med 12(2): e1001791 10.1371/journal.pmed.1001791 25710472 PMC4339836

[44] Groenheit R, Ghebremichael S, Pennhag A, Jonsson J, Hoffner S, Couvin D, et al (2012) *Mycobacterium tuberculosis* strains potentially involved in the TB epidemic in Sweden a century ago. PLoS One 7(10):e46848 10.1371/journal.pone.0046848 23056484 PMC3466202

[45] Douglas JT, Qian L, Montoya JC, Musser JM, Van Embden JDA, Van Soolingen D, et al (2003) Characterization of the Manila Family of *Mycobacterium tuberculosis*. J Clin Microbiol. 41(6):2723–6. 10.1128/JCM.41.6.2723-2726.2003 12791915 PMC156522

[46] Ferdinand S, Sola C, Chanteau S, Ramarokoto H, Rasolonavalona T, Rasolofo-Razanamparany V, et al (2005) A study of spoligotyping-defined *Mycobacterium tuberculosis* clades in relation to the origin of peopling and the demographic history in Madagascar. Infection, Genetics and Evolution. 5(4):340–8. 10.1016/j.meegid.2004.10.002 16168940

[47] Rahim Z, Zaman K, van der Zanden AGM, Möllers MJ, van Soolingen D, Raqib R, et al (2007) Assessment of Population Structure and Major Circulating Phylogeographical Clades of *Mycobacterium tuberculosis* Complex in Bangladesh Suggests a High Prevalence of a Specific Subclade of Ancient M. tuberculosis Genotypes. J Clin Microbiol. 45(11):3791–4. 10.1128/JCM.01247-07 17804653 PMC2168514

[48] Devi KR, Bhutia R, Bhowmick S, Mukherjee K, Mahanta J, Narain K (2015) Genetic Diversity of *Mycobacterium tuberculosis* Isolates from Assam, India: Dominance of Beijing Family and Discovery of Two New Clades Related to CAS1_Delhi and EAI Family Based on Spoligotyping and MIRU-VNTR Typing. PLoS ONE 10(12): e0145860 10.1371/journal.pone.0145860 26701129 PMC4689458

[49] Meehan CJ, Moris P, Kohl TA, Pečerska J, Akter S, Merker M, et al (2018) The relationship between transmission time and clustering methods in *Mycobacterium tuberculosis* epidemiology. EBioMedicine, 37:410–416. 10.1016/j.ebiom.2018.10.013 30341041 PMC6284411

[50] Rutaihwa LK, Sasamalo M, Jaleco A, Hella J, Kingazi A, Kamwela L, et al (2019) Insights into the genetic diversity of *Mycobacterium tuberculosis* in Tanzania. PLoS One 14(4):e0206334 10.1371/journal.pone.0206334 30978186 PMC6461268

[51] Conceição EC, Refregier G, Gomes HM, Olessa-Daragon X, Coll F, Ratovonirina NH, et al (2019) *Mycobacterium tuberculosis* lineage 1 genetic diversity in Pará, Brazil, suggests common ancestry with east-African isolates potentially linked to historical slave trade. Infection, Genetics and Evolution. 73:337–341. 10.1016/j.meegid.2019.06.001 31170529

[52] Chihota VN, Niehaus A, Streicher EM, Wang X, Sampson SL, Mason P, et al (2018) Geospatial distribution of *Mycobacterium tuberculosis* genotypes in Africa. PLoS ONE 13(8): e0200632 10.1371/journal.pone.0200632 30067763 PMC6070189

[53] Mogashoa T, Melamu P, Ley SD, Streicher EM, Iketleng T, Kelentse N, et al (2019) Genetic diversity of *Mycobacterium tuberculosis* strains circulating in Botswana. PLoS One 14(5):e0216306 10.1371/journal.pone.0216306 31063472 PMC6504092

