# Supplementary figures and images for: Two tales: Worldwide distribution of Central Asian (CAS) versus ancestral East-African Indian (EAI) lineages of Mycobacterium tuberculosis underlines a remarkable cleavage for phylogeographical, epidemiological and demographical characteristics
Source: PLoS One. 2019 Jul 12;14(7):e0219706. doi: 10.1371/journal.pone.0219706 (PMC6625721; doi:10.1371/journal.pone.0219706)

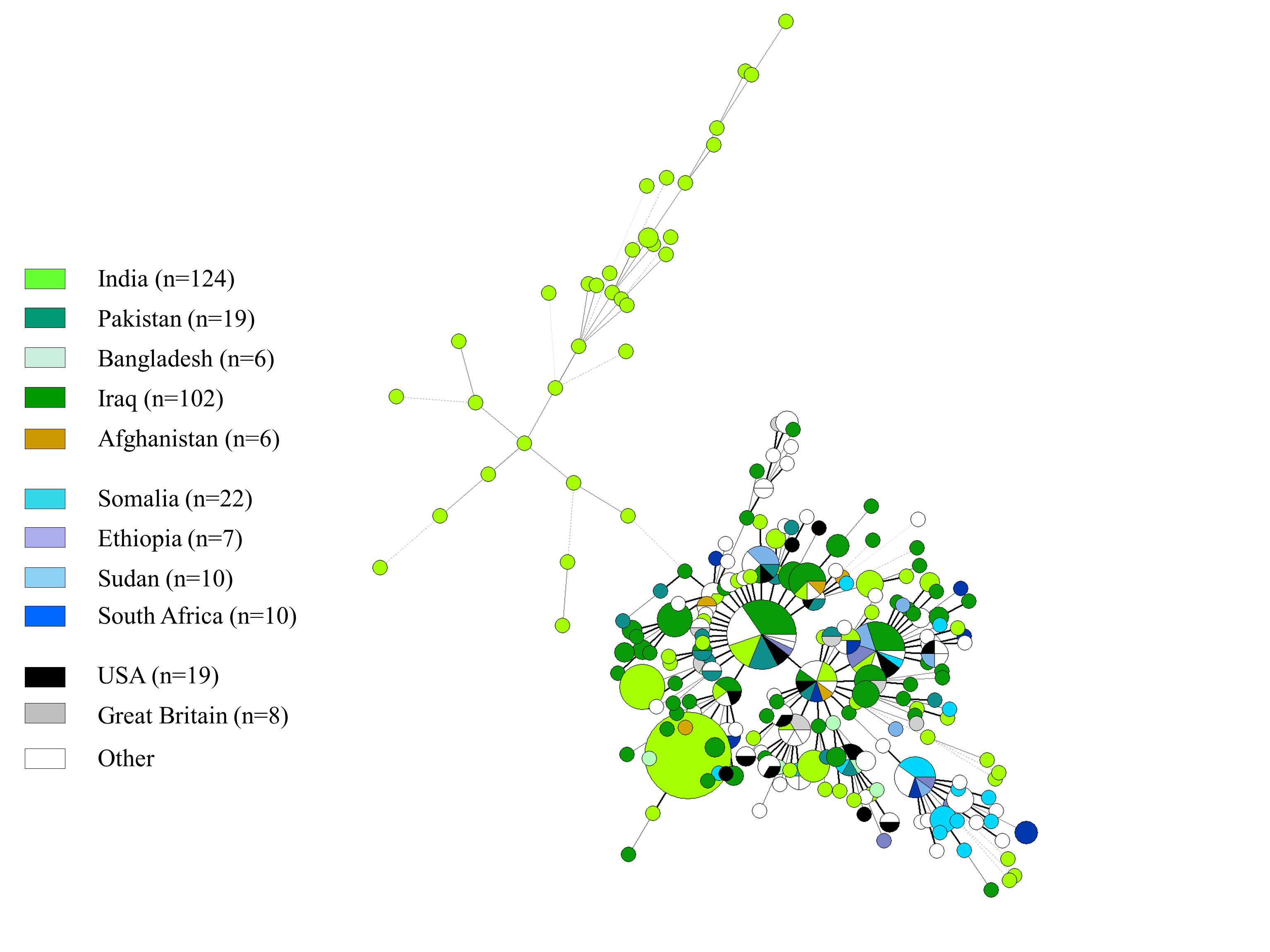

Supplement: S1 Fig — Size of nodes or complexity of lines are the same as in Figs 3 and 4. (TIF) [file pone.0219706.s001.tif]

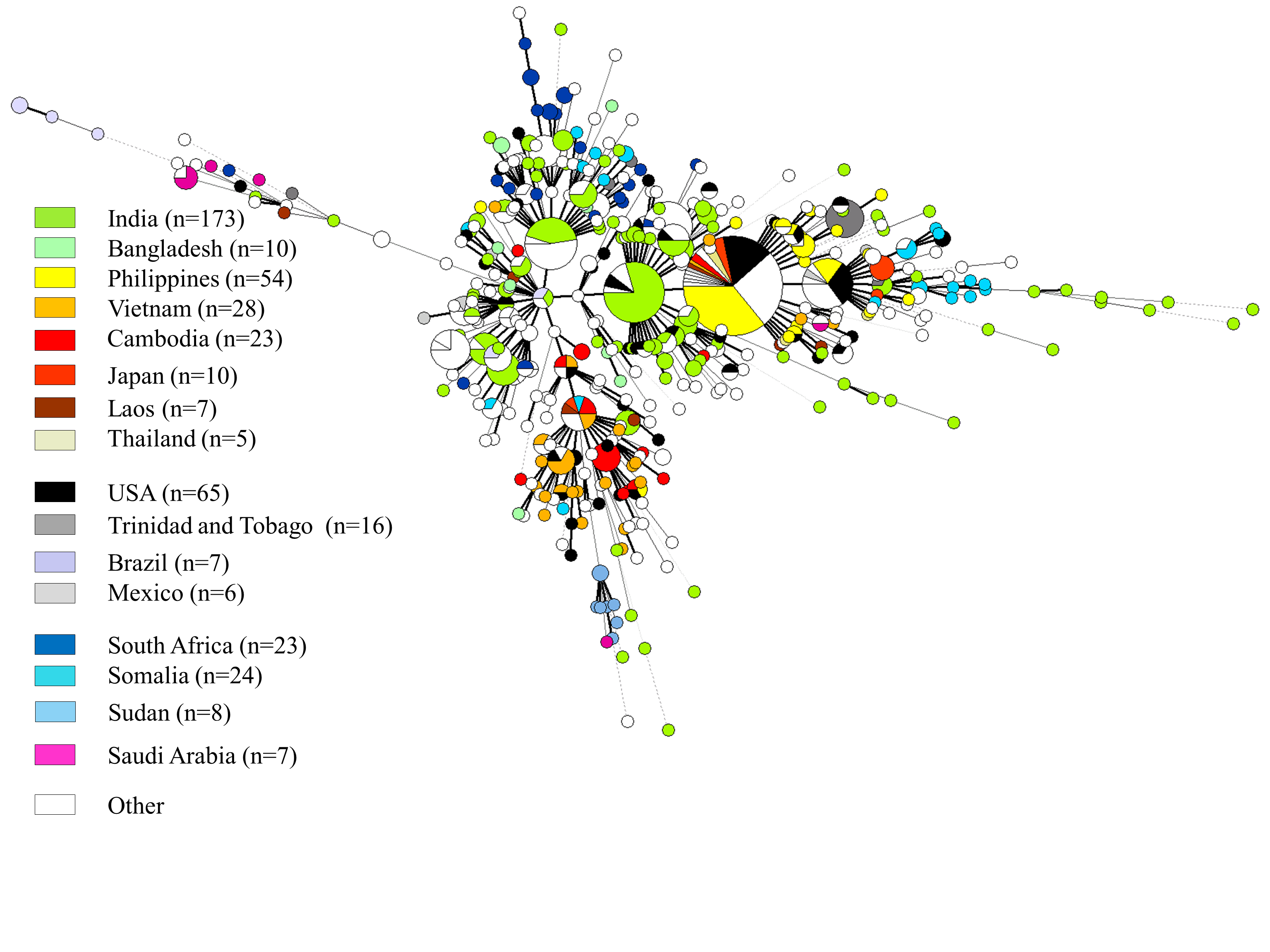

Supplement: S2 Fig — Size of nodes or complexity of lines are the same as in Figs 3 and 4. (TIF) [file pone.0219706.s002.tif]

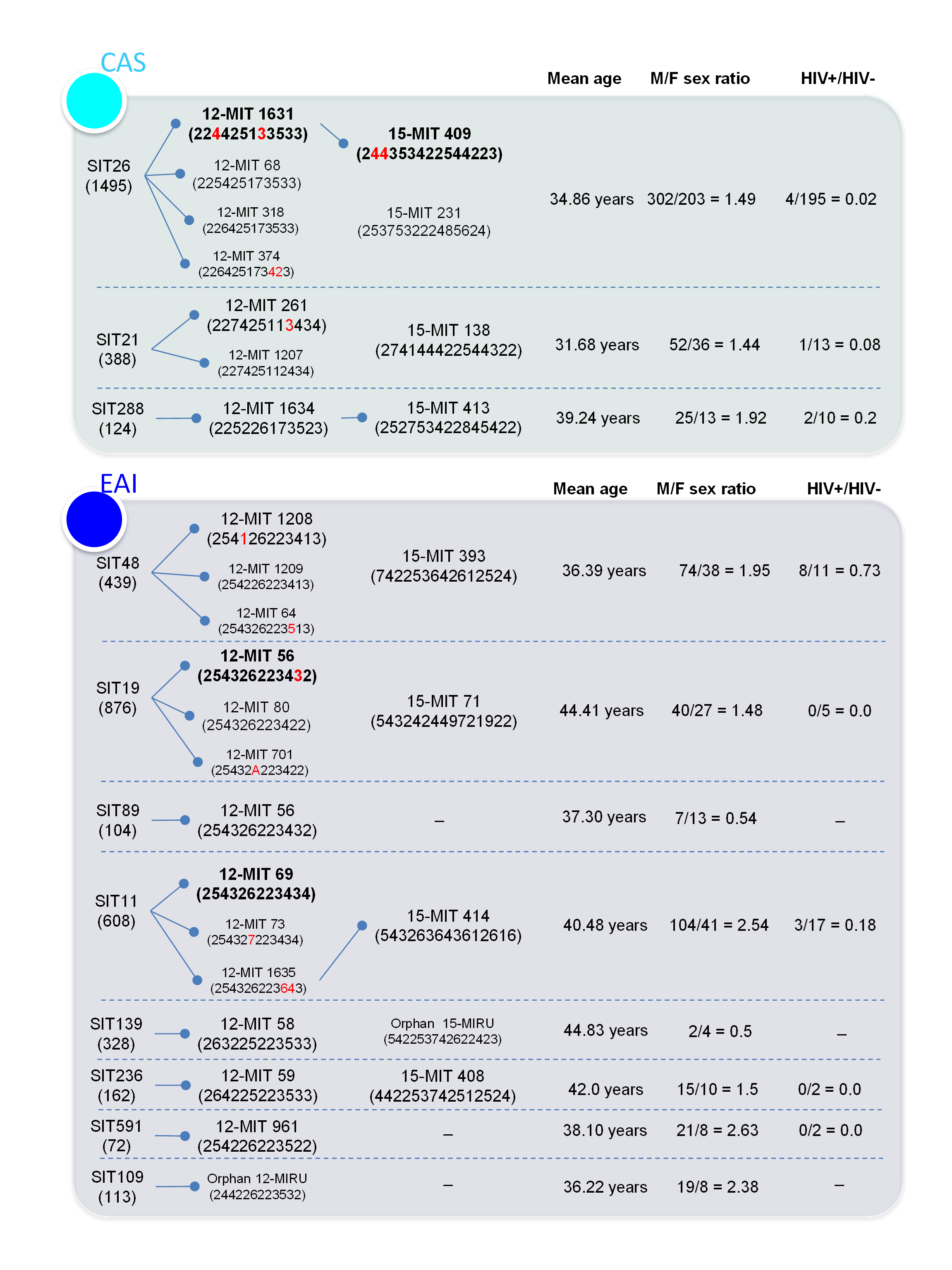

Supplement: S5 Fig — When SITs and/or MIRU-VNTRs genotypes were associated, a link was drawn. Size of associated MIRUs was roughly proportional to number of genotypes. Main changes between MIRU-VNTRs loci were shown in red. (TIF) [file pone.0219706.s005.tif]

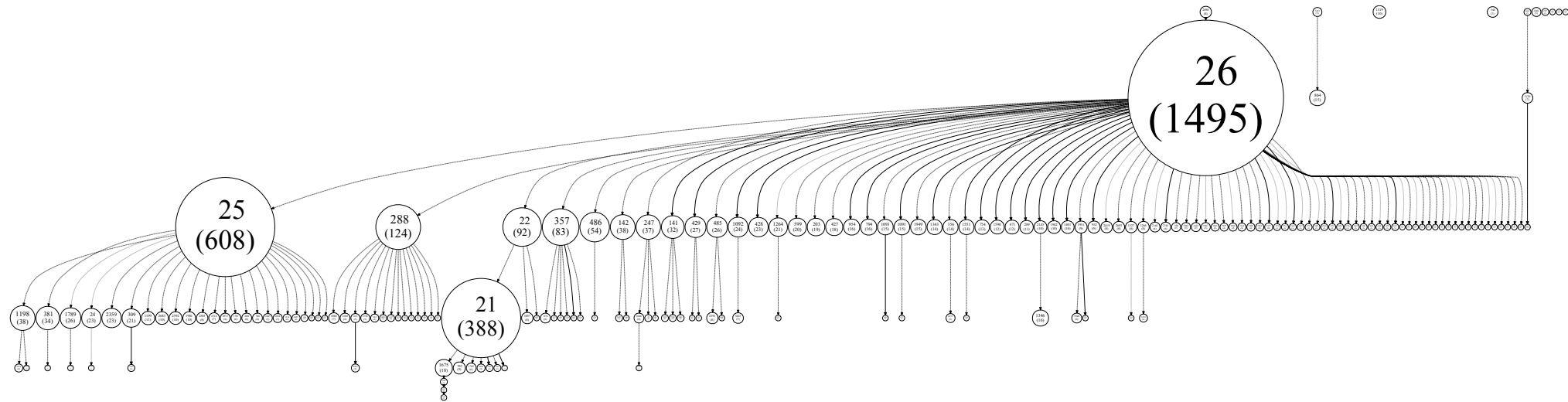

Supplement: S6 Fig — Each spoligotype pattern from the study is represented by a node with area size being proportional to the total number of isolates with that specific pattern (number shown in brackets under the SIT number). Changes (loss of spacers) are represented by directed edges between nodes, with the arrowheads pointing to descendant spoligotypes. In this representation, the heuristic used selects a single inbound edge with a maximum weight using a Zipf model. Solid black lines link patterns that are very similar, i.e., loss of one spacer only (maximum weigh being 1.0), while dashed lines represent links of weight comprised between 0.5 and 1, and dotted lines a weight less than 0.5. (PDF) [file pone.0219706.s006.pdf]

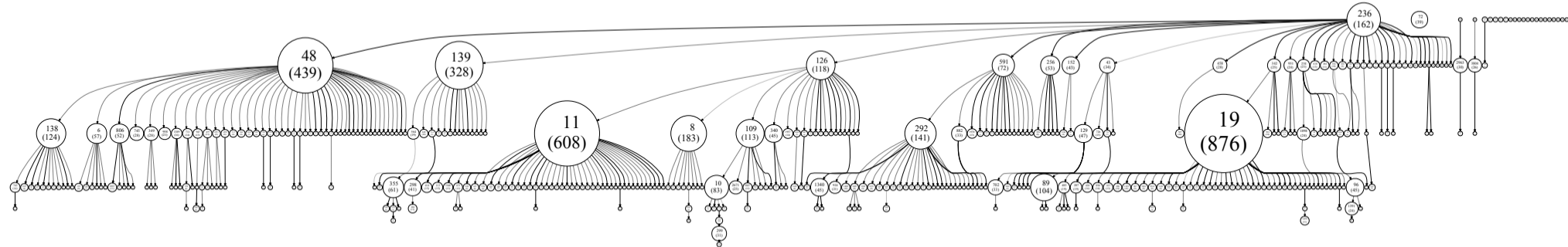

Supplement: S7 Fig — Each spoligotype pattern from the study is represented by a node with area size being proportional to the total number of isolates with that specific pattern (number shown in brackets under the SIT number). Changes (loss of spacers) are represented by directed edges between nodes, with the arrowheads pointing to descendant spoligotypes. In this representation, the heuristic used selects a single inbound edge with a maximum weight using a Zipf model. Solid black lines link patterns that are very similar, i.e., loss of one spacer only (maximum weigh being 1.0), while dashed lines represent links of weight comprised between 0.5 and 1, and dotted lines a weight less than 0.5. (PDF) [file pone.0219706.s007.pdf]

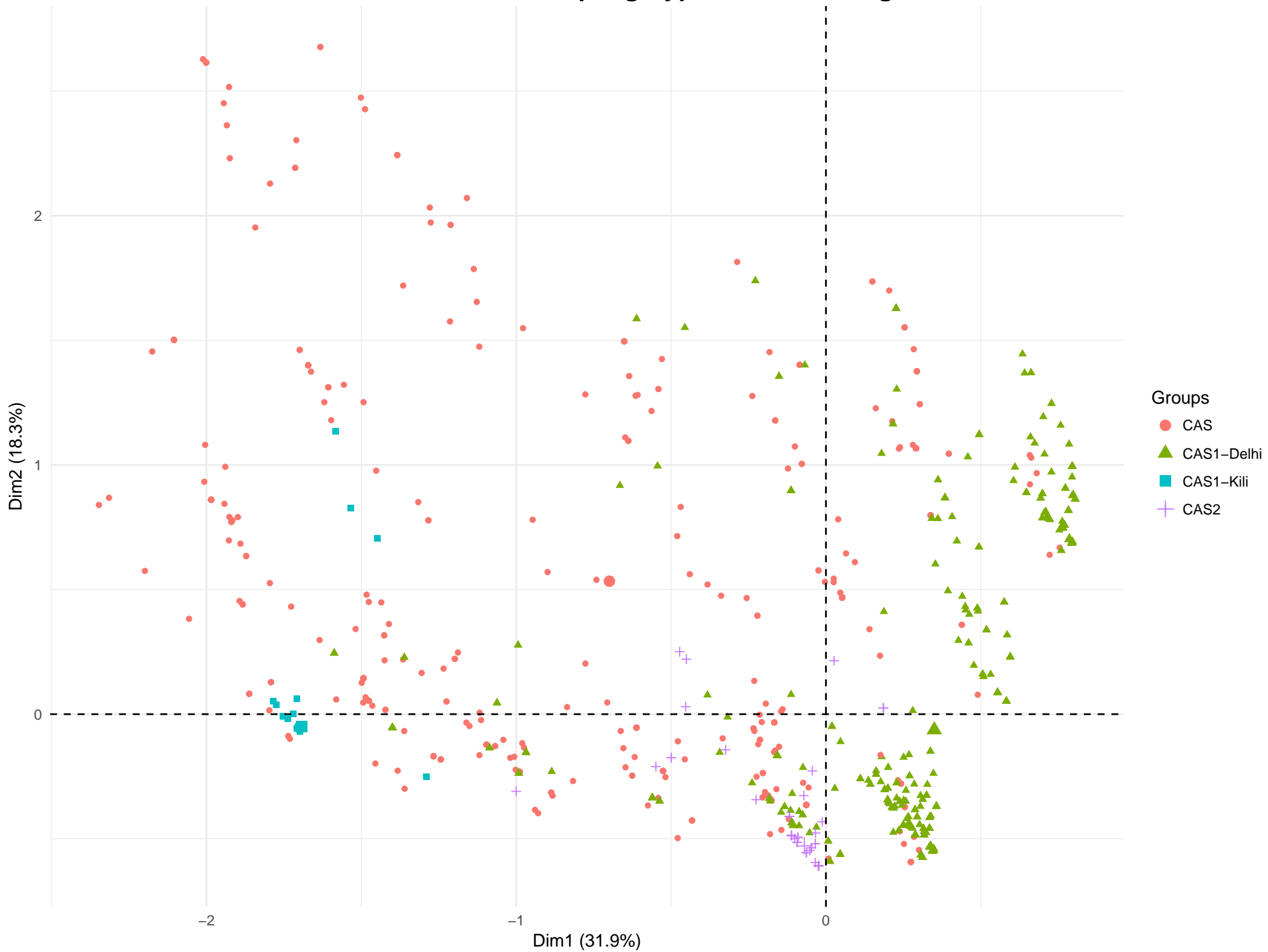

Supplement: S9 Fig — (PDF) [file pone.0219706.s009.pdf]

### PCA CAS spoligotypes vs. country of origin

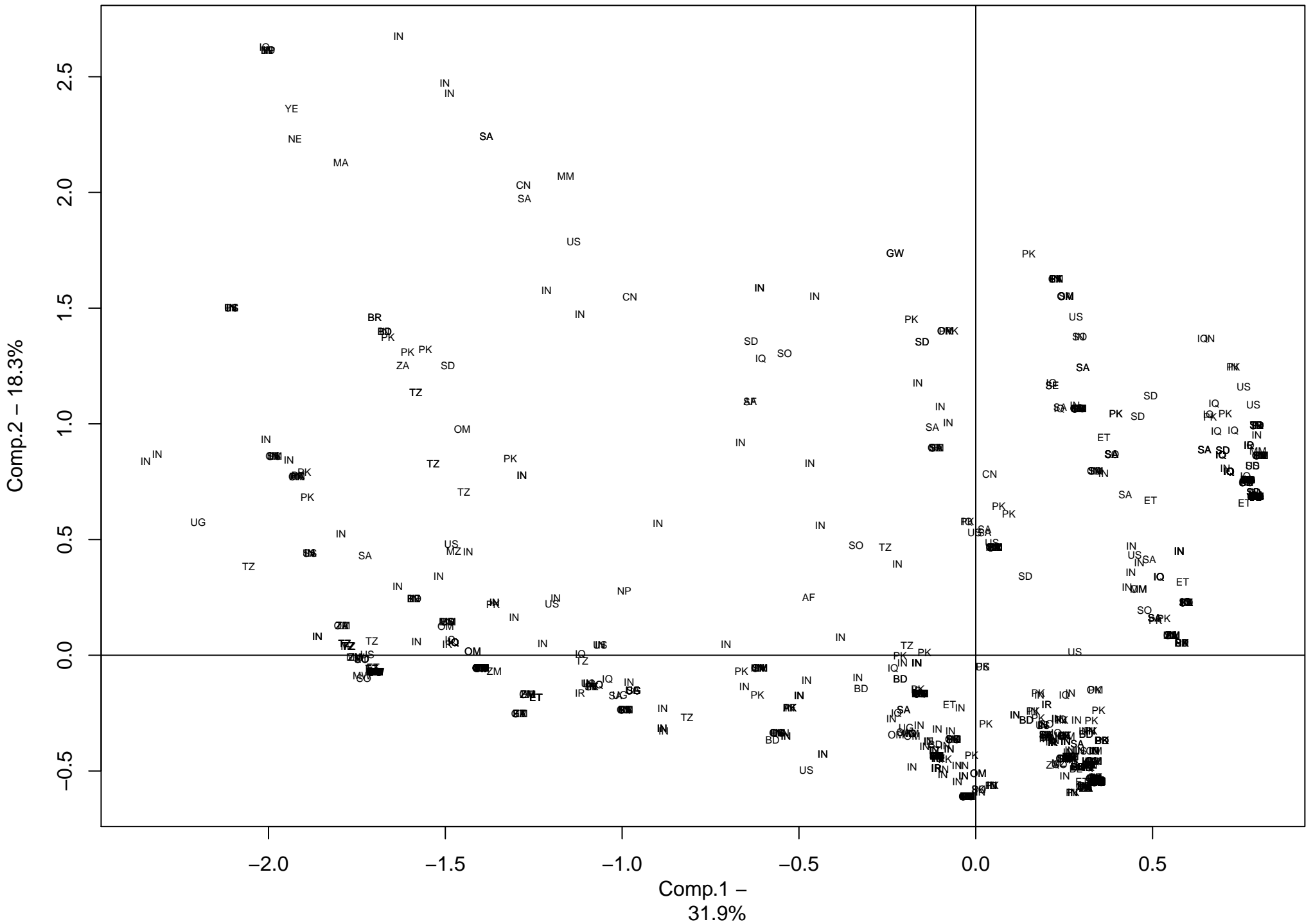

Supplement: S10 Fig — (PDF) [file pone.0219706.s010.pdf]

Individuals – PCA

PCA EAI spoligotypes vs. sublineages

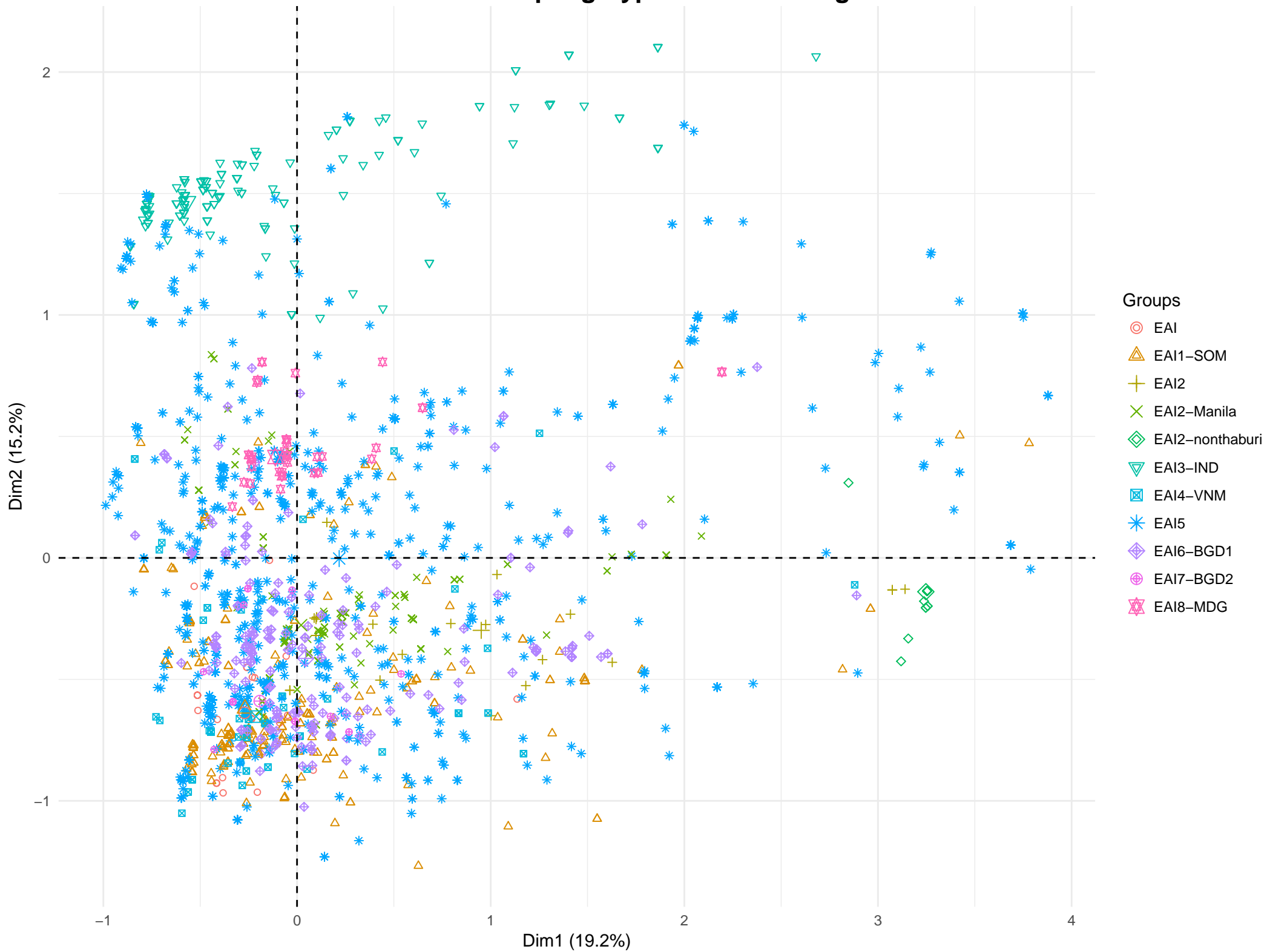

Supplement: S11 Fig — (PDF) [file pone.0219706.s011.pdf]

### PCA EAI spoligotypes vs. country of origin

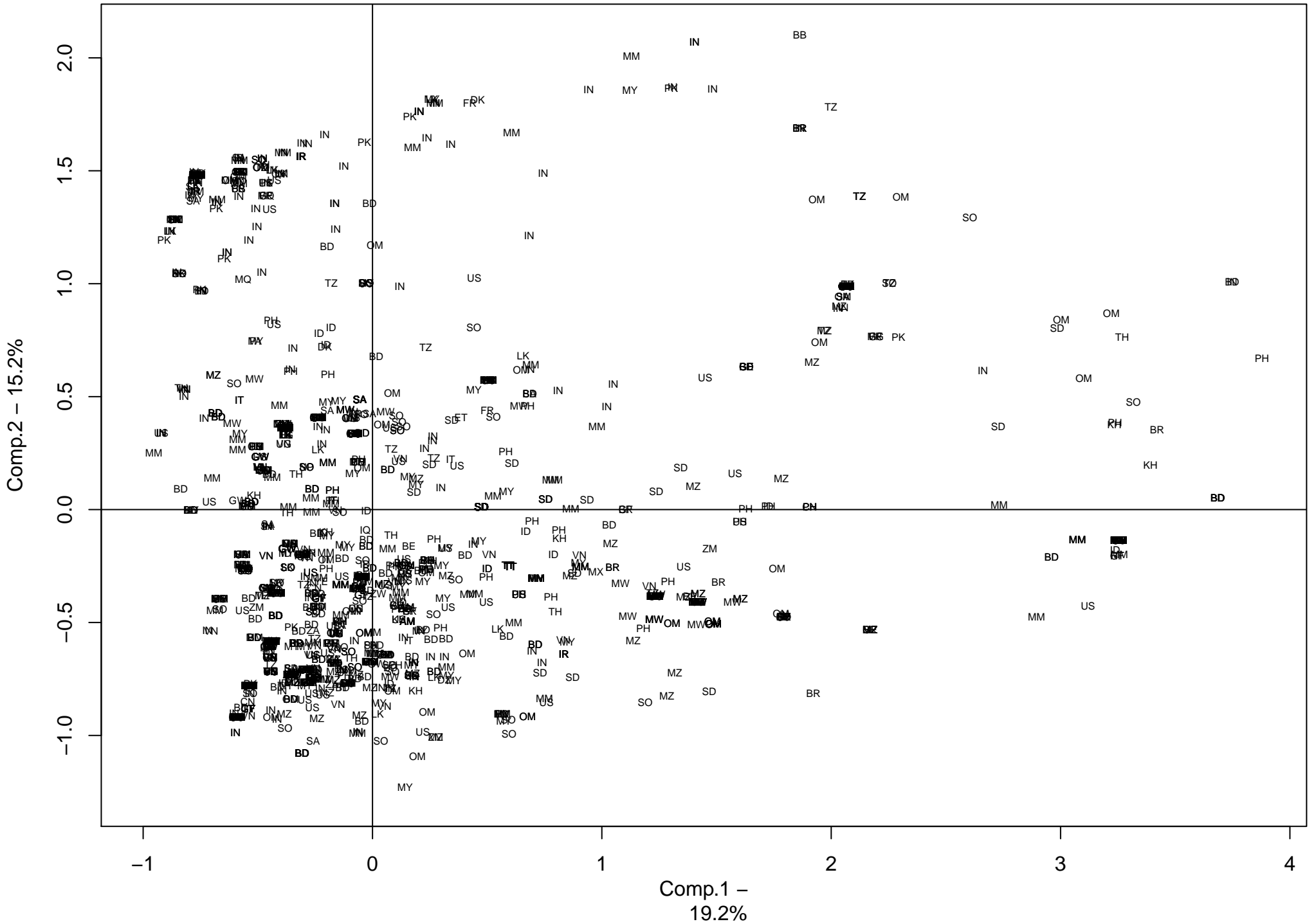

Supplement: S12 Fig — (PDF) [file pone.0219706.s012.pdf]

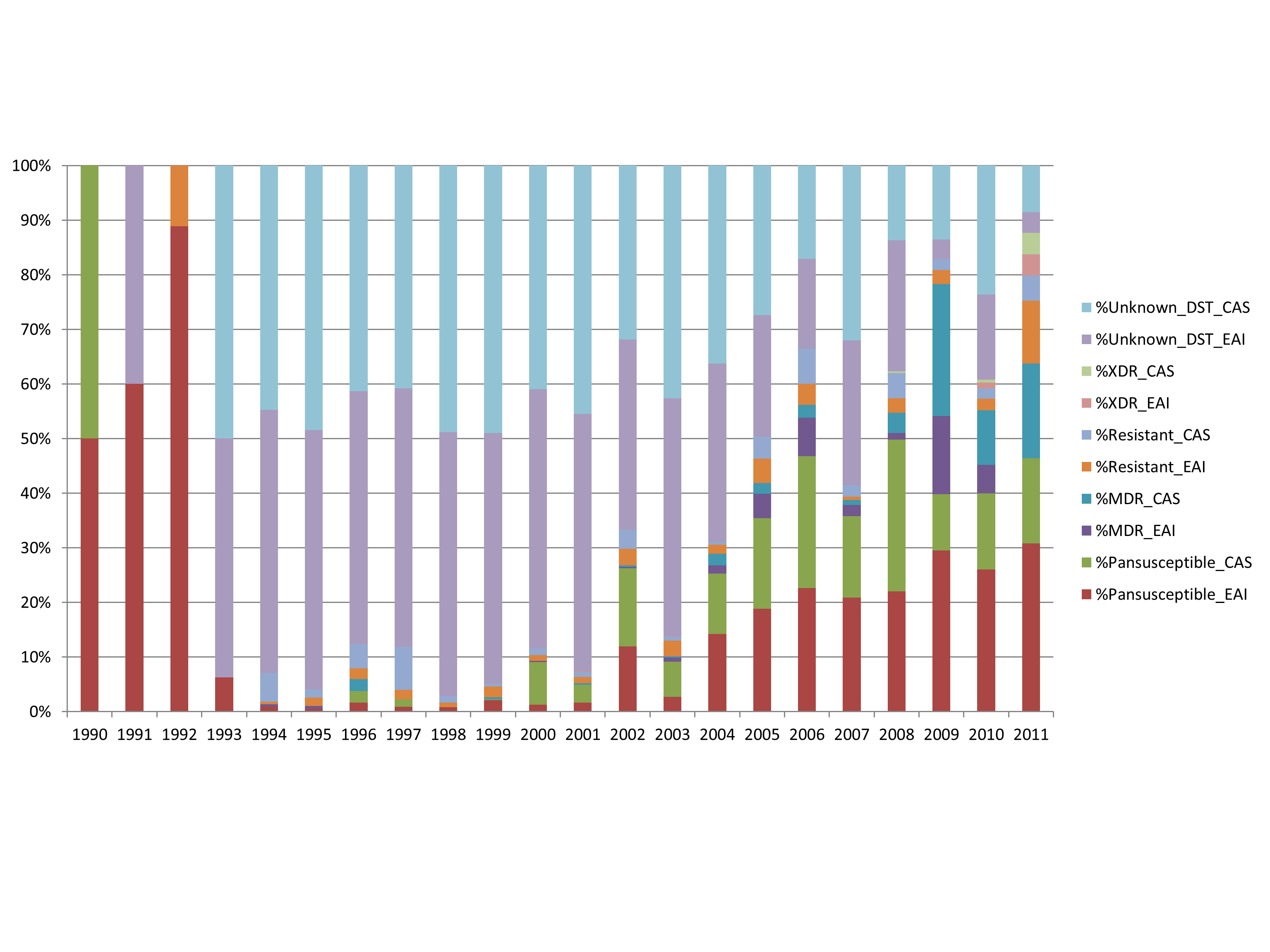

Supplement: S13 Fig — (TIF) [file pone.0219706.s013.tif]
