# Supplementary material for: Two tales: Worldwide distribution of Central Asian (CAS) versus ancestral East-African Indian (EAI) lineages of Mycobacterium tuberculosis underlines a remarkable cleavage for phylogeographical, epidemiological and demographical characteristics
Source: PLoS One. 2019 Jul 12;14(7):e0219706. doi: 10.1371/journal.pone.0219706 (PMC6625721; doi:10.1371/journal.pone.0219706)

Percentage of SIT 22

Pattern Value: 703777400001771

Spoligotype Description: ■■■□□□□■■■■■■■■■■■■□□□□□□□□□□□□□□□□■■■■■■■■

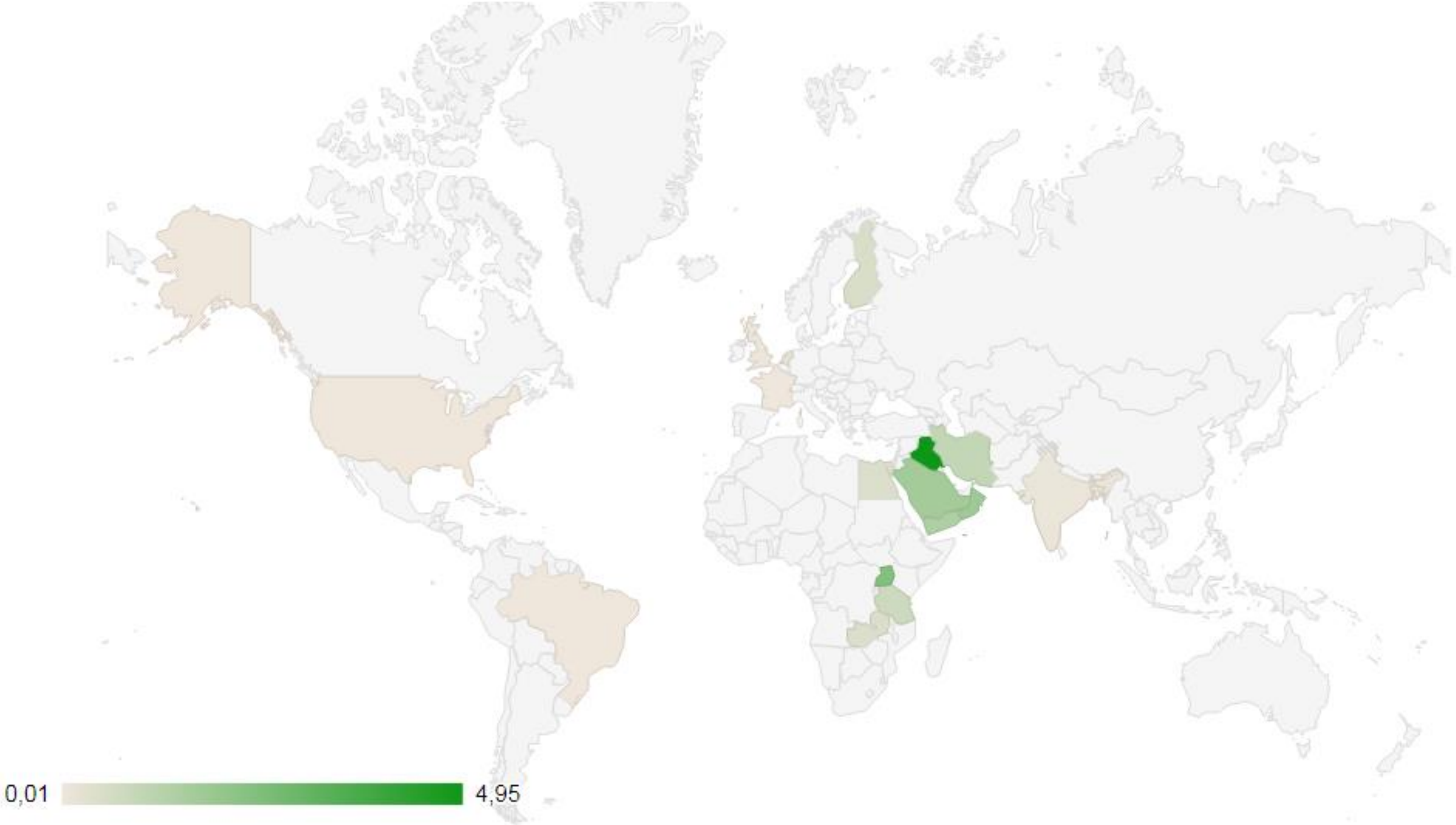

Percentage of SIT 25

Pattern Value: 703777740003171

Spoligotype Description: ■■■□□□□■■■■■■■■■■■■■■■□□□□□□□□□□□□■■□□■■■■■

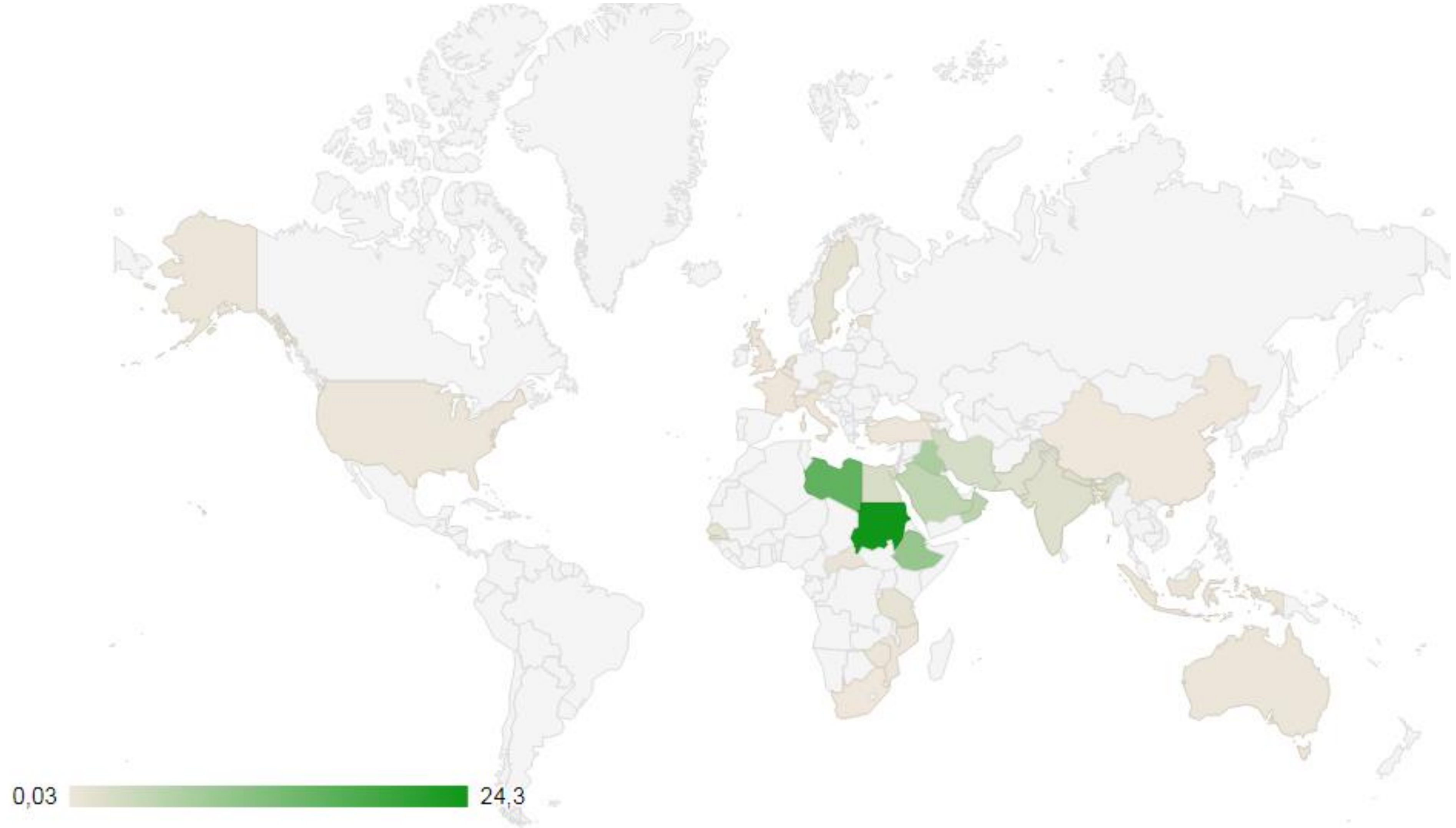

Percentage of SIT 292

Pattern Value: 777777757413371

Spoligotype Description: ■■■■■■■■■■■■■■■■■■■■■■□■■■■■□□□□■□■■□■■■■■■

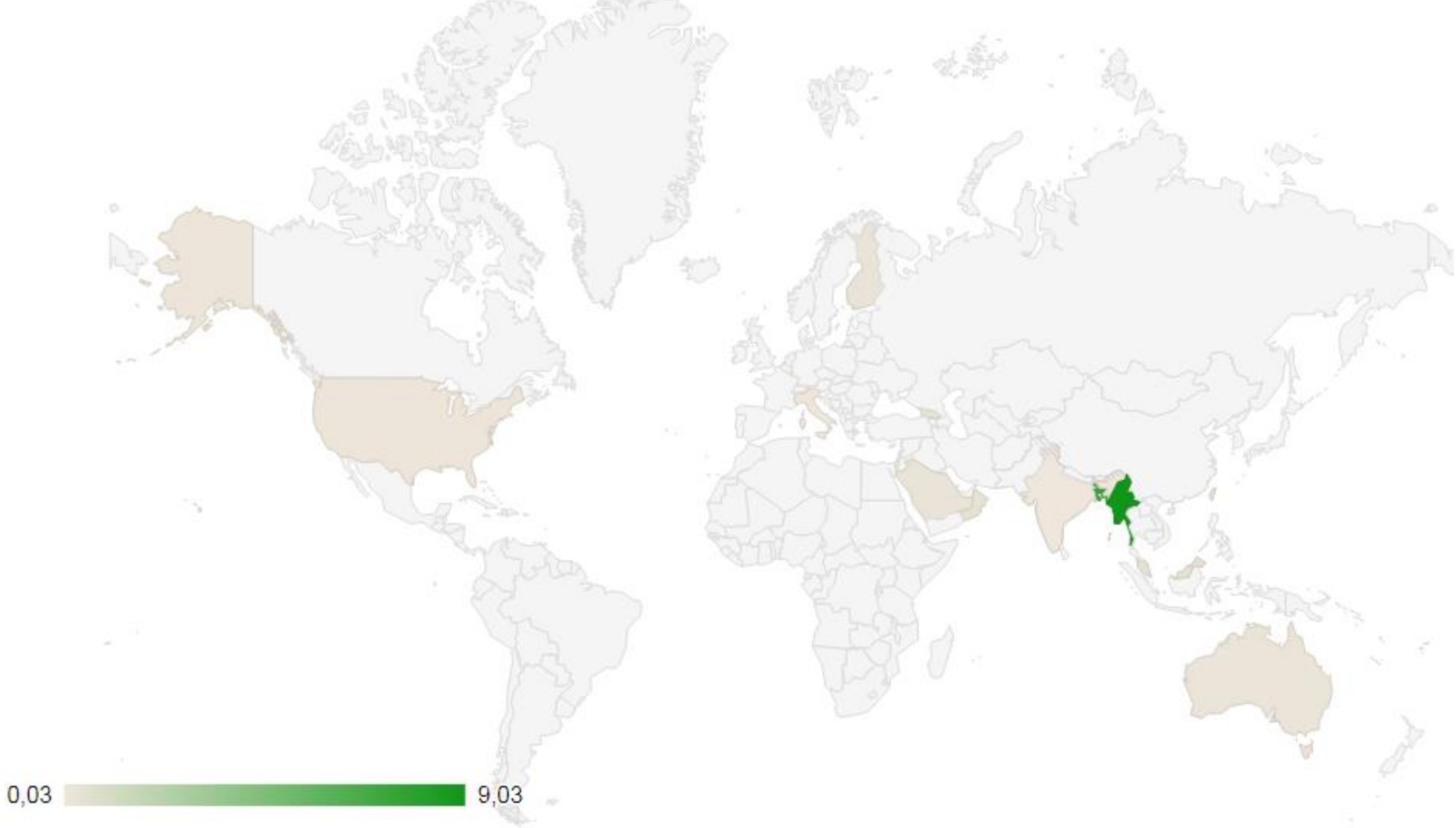

Percentage of SIT 1340

Pattern Value: 777775757413371

Spoligotype Description: ■■■■■■■■■■■■■■■■□■■■■■□■■■■■□□□□■□■■□■■■■■■

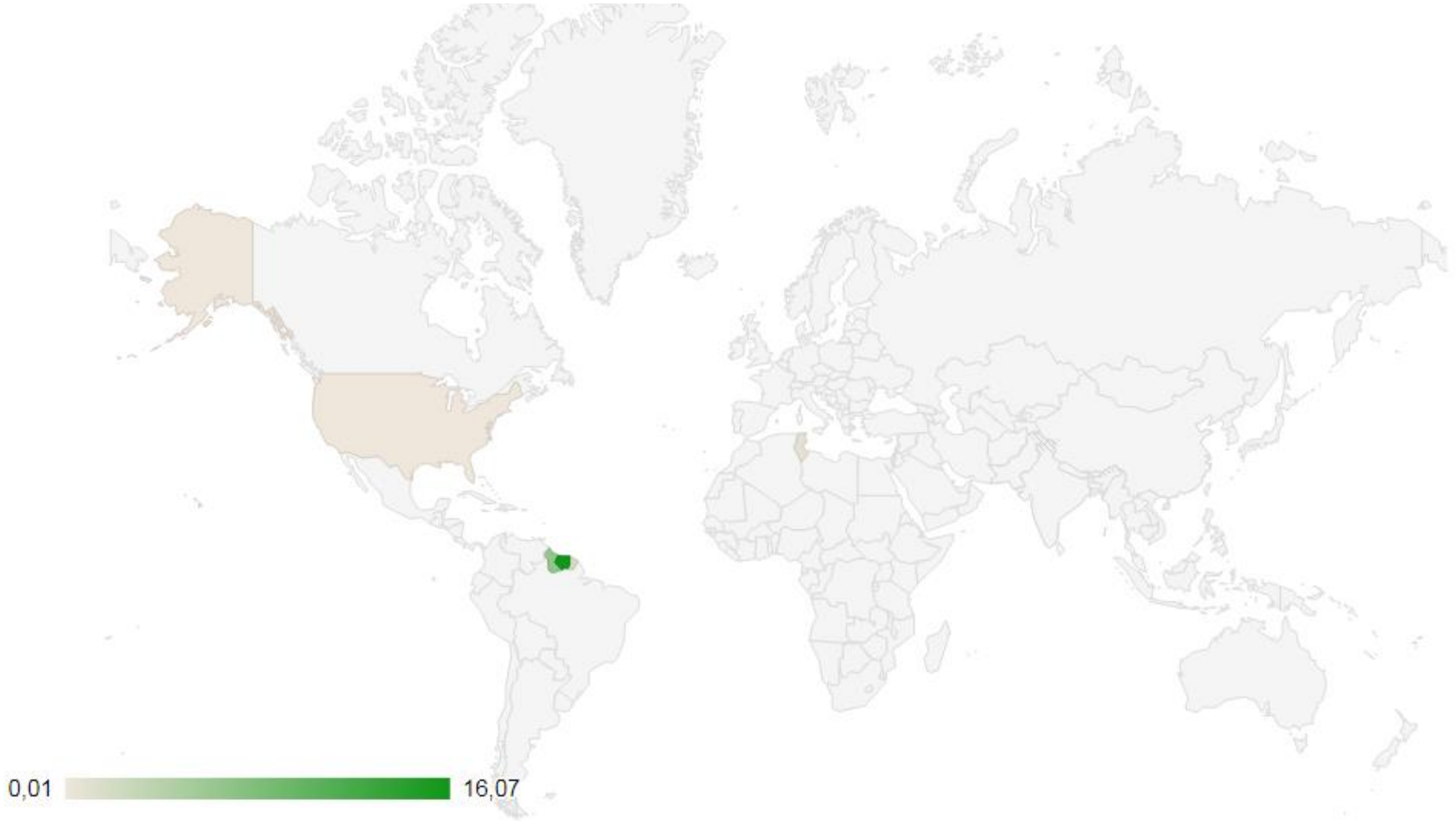

Supplement: S8 Fig — (PDF) [file pone.0219706.s008.pdf]
